# Feedback-Driven Assembly of the Axon Initial Segment

**DOI:** 10.1016/j.neuron.2019.07.029

**Published:** 2019-10-23

**Authors:** Amélie Fréal, Dipti Rai, Roderick P. Tas, Xingxiu Pan, Eugene A. Katrukha, Dieudonnée van de Willige, Riccardo Stucchi, Amol Aher, Chao Yang, A.F. Maarten Altelaar, Karin Vocking, Jan Andries Post, Martin Harterink, Lukas C. Kapitein, Anna Akhmanova, Casper C. Hoogenraad

**Affiliations:** 1 Cell Biology, Department of Biology, Faculty of Science, Utrecht University, Padualaan 8, 3584 CH Utrecht, the Netherlands; 2 Department of Axonal Signaling, Netherlands Institute for Neuroscience, Royal Netherlands Academy of Arts and Sciences, Meibergdreef 47, 1105 BA Amsterdam, the Netherlands; 3 Biomolecular Mass Spectrometry and Proteomics, Bijvoet Center for Biomolecular Research, Utrecht Institute for Pharmaceutical Sciences and the Netherlands Proteomics Center, Utrecht University, Padualaan 8, 3584 CH Utrecht, the Netherlands; 4 Department of Neuroscience, Genentech, Inc., South San Francisco, CA 94080, USA

**Keywords:** axon initial segment, axonal transport, microtubules, endocytosis

## Abstract

The axon initial segment (AIS) is a unique neuronal compartment that plays a crucial role in the generation of action potential and neuronal polarity. The assembly of the AIS requires membrane, scaffolding, and cytoskeletal proteins, including Ankyrin-G and TRIM46. How these components cooperate in AIS formation is currently poorly understood. Here, we show that Ankyrin-G acts as a scaffold interacting with End-Binding (EB) proteins and membrane proteins such as Neurofascin-186 to recruit TRIM46-positive microtubules to the plasma membrane. Using *in vitro* reconstitution and cellular assays, we demonstrate that TRIM46 forms parallel microtubule bundles and stabilizes them by acting as a rescue factor. TRIM46-labeled microtubules drive retrograde transport of Neurofascin-186 to the proximal axon, where Ankyrin-G prevents its endocytosis, resulting in stable accumulation of Neurofascin-186 at the AIS. Neurofascin-186 enrichment in turn reinforces membrane anchoring of Ankyrin-G and subsequent recruitment of TRIM46-decorated microtubules. Our study reveals feedback-based mechanisms driving AIS assembly.

## Highlights

• Ankyrin-G in complex with EBs recruits microtubule bundles to the plasma membrane
• TRIM46 is a rescue factor that forms stable parallel microtubule bundles
• TRIM46-bound microtubules direct Neurofascin-186 trafficking to the proximal axon
• Ankyrin-G controls Neurofascin-186 retention in the axon initial segment

## Teaser

Fréal et al. report the molecular mechanisms involved in axon initial segment (AIS) assembly. This study describes in detail how feedback-driven coupling between AIS membrane proteins and axonal microtubules allows for the formation and maintenance of a functional AIS.

## Introduction

The axon initial segment (AIS) is a specialized membrane-associated structure at the base of the axon that generates and shapes the action potential before it is propagated along the axon (Kole and Stuart, 2008). The AIS also functions as a boundary between the somatodendritic and axonal compartments to help maintain neuron polarity (Rasband, 2010). AIS components form a diffusion barrier segregating the somatodendritic and axonal membrane proteins and act as a cytoplasmic filter regulating cargo transport into the proximal axon (Leterrier, 2018). The AIS contains a concentration of voltage-gated ion channels and cell adhesion molecules that are anchored by a submembranous layer of scaffolds, extending from the plasma membrane to the underlying microtubule (MT) cytoskeleton. However, how the AIS is formed and maintained is still poorly understood.

In electron microscopy (EM) studies, the AIS is characterized by an ∼50-nm-thick submembranous undercoat lining the axonal plasma membrane and closely spaced bundles of 3–10 MTs also referred to as MT fascicles (Palay et al., 1968, Peters et al., 1968). The central component of the AIS submembrane complex is Ankyrin-G (ANK3), with a long isoform of 480 kDa (480AnkG) (Fréal et al., 2016, Jenkins et al., 2015b). Most of the AIS membrane proteins are directly recruited and concentrated by AnkG, including voltage-gated sodium (Nav) and potassium (Kv) channels and adhesion molecules, such as the 186-kDa isoform of Neurofascin (NF186) (Leterrier, 2018). The submembrane localization of AnkG depends on the association with AIS membrane proteins and palmitoylation of its amino-terminal domain (He et al., 2012, Le Bras et al., 2014, Leterrier et al., 2015, Leterrier et al., 2017). Depletion of 480AnkG not only prevents the accumulation of membrane proteins in the AIS but also impairs MT organization in the proximal axon (Fréal et al., 2016, Hedstrom et al., 2008, Sobotzik et al., 2009). The 480AnkG carboxy-terminal domain extends ∼35 nm into the cytoplasm and contains numerous SxIP motifs able to bind MTs via End-Binding (EB) proteins (Fréal et al., 2016, Leterrier et al., 2015). The interaction between EBs and AnkG is necessary for both AIS formation and maintenance (Fréal et al., 2016, Leterrier et al., 2011). These data highlight the importance of the association between the AIS submembrane complex and the underlying MT cytoskeleton. Still, the molecular mechanisms connecting axonal MT organization and AIS formation are incompletely understood.

Axon development depends on local MT stabilization and the formation of uniform MT bundles. The MT network at the AIS has specific properties compared to MTs in the rest of the neuron. AIS MTs were shown to be enriched in GTP-like tubulin (Nakata et al., 2011), and overall the AIS cytoskeleton and associated proteins are very stable, as shown by their high resistance to detergent extraction (Garrido et al., 2003, Sánchez-Ponce et al., 2012). The unique properties of AIS MTs are conferred by the presence of several MT-associated proteins (MAPs) that have been recently identified in the proximal axon, such as EB proteins (Nakata and Hirokawa, 2003), CAMSAP2 (Yau et al., 2014), MAP6 (Tortosa et al., 2017), MTCL-1 (Satake et al., 2017), MAP2 (Gumy et al., 2017), and TRIM46 (van Beuningen et al., 2015). TRIM46 belongs to the tripartite motif containing (TRIM) protein family of ubiquitin E3 ligases, but neither ubiquitin ligase activity nor substrates for TRIM46 have been reported (Meroni and Diez-Roux, 2005). Instead, TRIM46 associates with MTs in the proximal axon, where it forms plus-end-out MT bundles. TRIM46 is required for neuronal polarity and axon specification *in vitro* and *in vivo* (van Beuningen et al., 2015). TRIM46 is an early axonal marker because it localizes to the future axon before neuronal polarization and AIS assembly. Interestingly, expression of TRIM46 in heterologous cells induces the formation of bundles of closely spaced MTs linked by electron-dense cross-bridges, which closely resemble the axon-specific MT fascicles (Harterink et al., 2019, van Beuningen and Hoogenraad, 2016, van Beuningen et al., 2015). Defining the interplay between AnkG and TRIM46-induced MT fascicles may thus provide a molecular pathway for AIS assembly.

In this study, we used live-cell imaging in combination with biochemical and cell biological assays, as well as microscopy-based *in vitro* reconstitution assays to determine the mechanisms underlying AIS assembly. We show that plasma membrane-attached 480AnkG actively recruits and anchors MT bundles to the plasma membrane. TRIM46 generates uniform MT fascicles by selectively promoting growth of parallel-oriented MTs. Subsequently, TRIM46-decorated MT bundles locally concentrate AnkG and its associated protein NF186. The uniform MT organization drives efficient transport of NF186 to the AIS, where it interacts with AnkG to stably accumulate at the AIS. Our study reveals a feedback-driven mechanism for the assembly of the AIS.

## Results

### 480AnkG Recruits MTs to the Plasma Membrane

**Figure 1:**
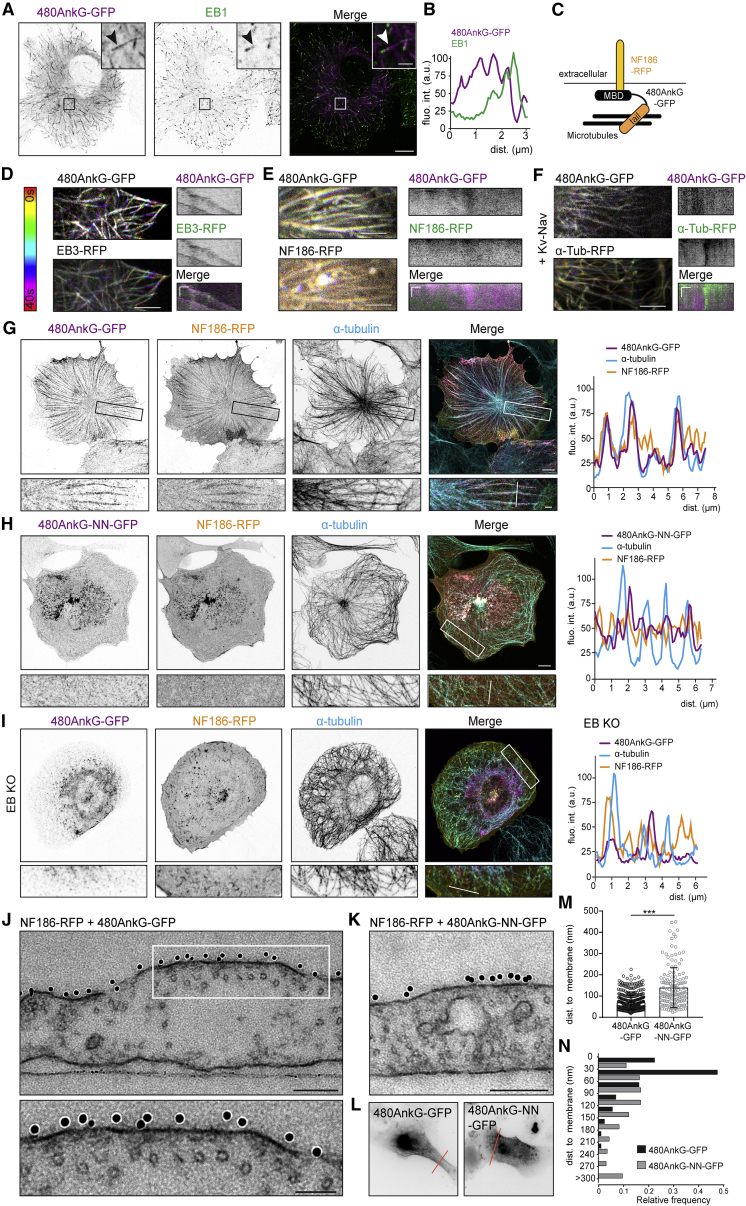
480AnkG Organizes MTs at the Plasma Membrane in an EB-Dependent Manner (A and B) COS-7 cell expressing 480AnkG-GFP, stained for GFP and EB1 (A). (B) shows fluorescence intensities along the comet pointed in the zoom. (C) Scheme showing 480AnkG-GFP targeted to the plasma membrane by NF186-RFP via its membrane-binding domain (MBD), while its tail domain anchors MTs. (D–F) Temporal-coded maximum projections from time-lapse imaging of COS-7 cells expressing 480AnkG-GFP with EB3-RFP (D), NF186-RFP (E), α-Tub-RFP (F). Representative kymographs and color-coded timescale is shown. (G and H) COS-7 cells co-expressing NF186-RFP together with 480AnkG-GFP (G) or 480AnkG-NN-GFP (H) and stained for α-tubulin. (I) EB1/2/3 KO U2OS cell co-expressing NF186-RFP and 480AnkG-GFP and stained for α-tubulin. (J–L) EM pictures of COS-7 cells co-expressing NF186-RFP with 480AnkG-GFP (J) or 480AnkG-NN-GFP (K) and immunogold labeled for extracellular NFasc. Fluorescent pictures of corresponding cells are shown in (L), and cutting sites are indicated with a line. (M and N) Distance between MTs and the plasma membrane (M) and corresponding frequency plot (N). Mann-Whitney test, p = 3.26^∗^10^–9^, n = 123 MTs for AnkG-GFP in N = 3 cells, n = 102 MTs for 480AnkG-NN-GFP in N = 2 cells. In (A) and (G–I), scale bars are 10 μm and 2 μm in the zoom. In (D–F), scale bars are 5 μm and on the corresponding kymographs: 1 μm (horizontal) and 15 s (vertical). For (J) and (K), scale bars are 200 and 100 nm in the zoom. See also Figure S1.

Our previous study showed that 480AnkG possesses numerous SxIP motifs (Figure S1A), which allow it to track MT plus-ends in an EB-dependent manner. Indeed, expression of 480AnkG-GFP in COS-7 cells revealed a comet-like distribution, which colocalized with EB1 (Figures 1A and 1B), and live imaging showed a robust MT plus-end tracking behavior (Figures 1D and S1B; Video S1). However, co-expression of 480AnkG-GFP together with one of its known AIS interacting membrane proteins, either NF186-RFP (Figures 1C, 1E, 1G, S1C, and S1D) or myc-Kv2.1-Nav1.2 (KvNav) (Bréchet et al., 2008) (Figures 1F and S1E), induced membrane recruitment of 480AnkG. When targeted to the plasma membrane by AIS membrane proteins, 480AnkG-GFP formed long linear stretches that stained positive for tubulin (Figure 1G). Live imaging revealed that membrane-attached 480AnkG-GFP no longer tracked MT-plus ends but was distributed along the MT lattice (Figures 1E and 1F), indicating that 480AnkG recruited MTs to the plasma membrane

Video S1. 480AnkG Colocalizes with EB3 and Tracks MT Plus-ends, Related to Figure 1 Time-lapse movie of a COS-7 cell co-expressing 480AnkG-GFP (magenta) with EB3-RFP (green). The movie consists of 121 frames recorded simultaneously in the two channels with 1 s interval between frames and 100 ms exposure time. Scale bar represents 5 μm.

The ability of 480AnkG to organize MT structures at the plasma membrane relies on the interaction with both MTs and AIS membrane proteins. The 480AnkG tail construct that interacts with EBs but does not bind to AIS membrane proteins (Fréal et al., 2016) behaved as a +TIP when co-expressed with NF186 (Figure S1F). Similarly, when 480AnkG-GFP was co-expressed with NF186 mutated in its AnkG-binding motif (FIGQY→ FIGQD) (Boiko et al., 2007, Zhang et al., 1998), 480AnkG-GFP still tracked MT-plus ends and did not redistribute along MT lattice (Figures S1G and S1H). Mutation of the 480AnkG palmitoylation residue (He et al., 2012) did not change the NF186-dependent membrane recruitment of 480AnkG in COS-7 cells (Figure S1I), nor its membrane association in days in vitro (DIV)3 neurons (Figure S1J). AnkG mutants that do not interact with EBs, such as 480AnkG-NN (in which 10 SxIP motifs are changed into SxNN, Figure S1A) or 270AnkG (Fréal et al., 2016), showed a diffuse cytoplasmic staining and did not recruit MTs to the plasma membrane in the presence of NF186-RFP (Figures 1H and S1K). Moreover, in U2OS cells in which EB1, EB2, and EB3 were stably knocked out (Yang et al., 2017) (Figures S2A and S2F), 480AnkG-GFP showed no accumulation at MT plus-ends (Figure S2A) and did not anchor MTs to the plasma membrane when co-expressed with NF186-RFP (Figures 1I versus S2B). The expression of EB3-RFP in these KO cells could rescue the MT plus-end localization of 480AnkG and its ability to anchor MTs at the membrane when co-expressed with KvNav (Figures S2C–S2E). These data suggest that the interaction with EBs is critical for the 480AnkG-dependent recruitment of MTs to the plasma membrane.

To further study 480AnkG’s ability to recruit MTs to the plasma membrane, we examined COS-7 cells co-expressing NF186-RFP together with either 480AnkG-GFP or 480AnkG-NN-GFP (Figures 1J–1L) by EM. Pre-embedding immunogold labeling of NF186 allowed us to resolve the position of the AIS membrane proteins at the plasma membrane (Figure S2J). Quantifications revealed that 480AnkG organizes MTs in close proximity to the plasma membrane (30–60 nm), whereas 480AnkG-NN did not induce any significant clustering of MTs (Figures 1M, 1N, and S2I). We also observed a dark membrane undercoating where NF186 was concentrated, as noticed in previous EM studies of the AIS (Palay et al., 1968). We then used gated stimulated emission depletion (gSTED) microscopy to observe MT position with respect to the cell cortex in COS-7 cells expressing various constructs and stained with phalloidin (Figures S2G and S2H). In agreement with the EM data, 480AnkG-GFP, but not 480AnkG-NN-GFP, induced a clear recruitment of MTs to the cortex in the presence of a membrane binding partner (Figures S2G and S2H, lower panels). Interestingly, neither 480AnkG-GFP, which can bind to MT plus ends, nor the diffuse 480AnkG-NN-GFP could induce such a MT organization on their own (Figures S2G and S2H, upper panels).

**Figure 2:**
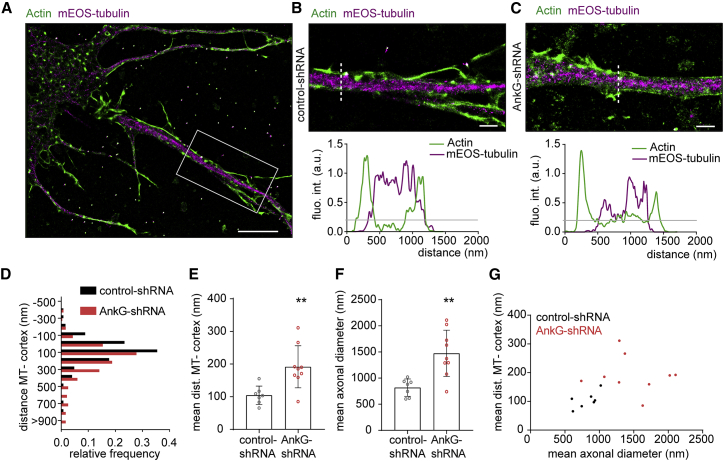
AnkG Anchors MTs Close to the Axonal Membrane (A–C) Single-molecule localization microscopy reconstructions of DIV4 hippocampal neurons transfected at DIV0 with mEOS-tubulin and a control- (A and B) or AnkG-shRNA (C). Intensity profiles along the indicated lines are shown. (D) Distribution of the MT-cell cortex distances along the proximal axons of control neurons or neurons lacking AnkG. Control: 124 measures from 7 neurons, AnkG-shRNA: 234 measures, N = 2, n = 9 neurons. (E–G) Mean MT-cell cortex distance (E), axonal diameter (F), and mean axonal diameter as a function of MT-cell cortex distance (G) of neurons transfected with a control-shRNA or AnkG-shRNA. Unpaired t tests, p = 0.058 in (E), p = 0.026 in (F) and in (G); Pearson’s correlation coefficients are p = 0.10 (control) and p = 0.72 (AnkG-shRNA). Scale bars are 5 μm in (A) and 1 μm in (B) and (C).

To assess whether AnkG is also capable of targeting MTs to the membrane vicinity in axons, we used single-molecule localization microscopy (SMLM) to resolve the distance between MTs and the plasma membrane in control neurons (Figures 2A and 2B) or neurons lacking AnkG (Figure 2C). As most of the AIS membrane proteins are lost upon depletion of AnkG, we imaged actin to determine the position of the cell cortex (Kiuchi et al., 2015, Schatzle et al., 2018). We observed that, in neurons lacking AnkG, the MT-cortex distances were shifted to higher values (Figure 2D) and the mean MT-cortex distance significantly increased (Figure 2E). As previously reported (Hedstrom et al., 2008), neurons transfected with AnkG-short hairpin RNA (shRNA) had a larger axonal diameter (Figure 2F). However, this was unlikely to be the cause of the increased MT-cortex distance, because the mean axonal diameter and the mean MT-cortex distance did not correlate (Figure 2G). Together, our data indicate that AnkG recruits MTs to the plasma membrane at the AIS.

### 480AnkG Recruits TRIM46-Decorated Parallel MT Bundles to the Plasma Membrane

**Figure 3:**
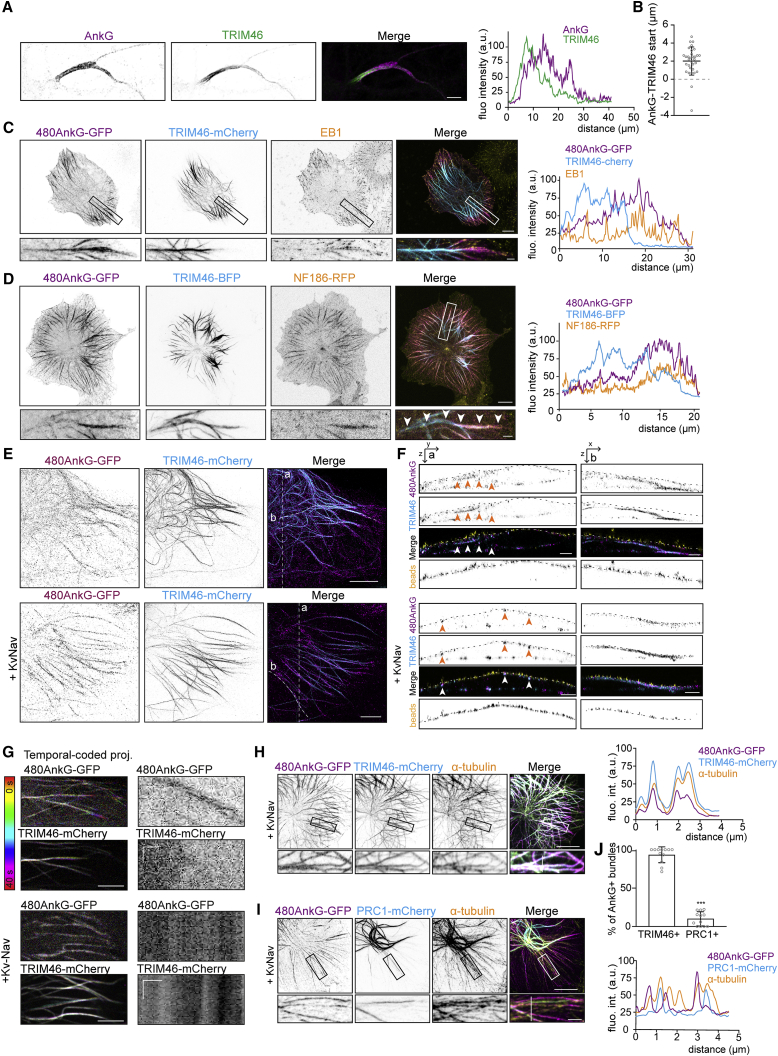
480AnkG Anchors TRIM46-Decorated MT Fascicles to the Plasma Membrane (A and B) Proximal region of a DIV14 hippocampal neuron stained for TRIM46 and AnkG (A). The difference between AnkG and TRIM46 start positions was measured in (B) for 32 neurons. (C and D) COS-7 cell expressing 480AnkG-GFP with TRIM46-mCherry (C) or TRIM46-BFP and NF186-RFP (D). (E and F) Expanded COS-7 cells (E) transfected with indicated constructs and stained for GFP and TRIM46. (F) shows average projections of resliced z stacks along transversal (left panel, a) or longitudinal (right panel, b) lines of 0.25 μm thickness. Arrowheads point at AnkG- and TRIM46-positive bundles. (G) Temporal-coded maximum projections from time-lapse imaging of COS-7 cells expressing indicated constructs. (H–J) COS-7 cell expressing 480AnkG-GFP and KvNav with TRIM46-mCherry (H) or PRC1-mCherry (I) and stained for α-tubulin. The percentage of positive membrane 480AnkG-GFP bundles is shown in (J). In (A), scale bar is 5 μm, in (C), (D), (H), and (I) scale bars are 10 μm and 2 μm in the zooms. In (E), scale bars are 5 μm and 2 μm in the z sections, and in the kymographs in (G) they represent 1 μm (horizontal) and 15 s (vertical). See also Figures S2 and S3.

In cultured primary neurons, TRIM46-labeled MT bundles located at the beginning of the axon as previously described (van Beuningen et al., 2015) and overlapped with AnkG, which localized to the axonal sub-membrane region (Figures 3A–3C and S3A). Depletion of TRIM46 led to a significant reduction in AnkG intensity at the AIS, whereas specific accumulation of TRIM46 in the proximal axon strongly relied on AnkG (van Beuningen et al., 2015) (Figures S3B–S3D). In COS-7 cells, when co-expressed with TRIM46-mCherry in the absence of membrane partners, 480AnkG-GFP was no longer observed at MT plus-ends only but instead coincided with TRIM46 along the MT lattice (Figure 3C). Interestingly, while 480AnkG-GFP and TRIM46-mCherry signals overlapped, they showed a spatial organization similar to that of the AIS, with TRIM46 being accumulated proximally along the MT bundle, while 480AnkG was enriched more distally toward the MT plus-ends (Figure 3C). Expression of other MAPs, such as anti-parallel MT bundler, PRC1-mCherry (Loïodice et al., 2005), or parallel MT bundler TRIM36-mCherry, a close homolog of TRIM46 that does not accumulate at the AIS (van Beuningen et al., 2015), did not recruit 480AnkG to MT bundles (Figures S3E and S3F), suggesting that 480AnkG selectively interacts with TRIM46-decorated MT bundles. Therefore, we tested whether 480AnkG specifically recruits TRIM46-labeled MTs to the plasma membrane. Co-expression of 480AnkG-GFP, NF186-RFP, and TRIM46-BFP in COS-7 cells revealed that MT bundles recruited by 480AnkG are labeled with TRIM46 (Figure 3D), suggesting that 480AnkG targets TRIM46-decorated parallel MT bundles to the plasma membrane. We used expansion microscopy (Chen et al., 2015, Tillberg et al., 2016) and gSTED microscopy to resolve the position of TRIM46-bundles in COS-7 cells expressing TRIM46-mCherry together with 480AnkG-GFP with or without KvNav (Figures 3E, 3F, and S3G–S3I). We observed that TRIM46 co-expressed with 480AnkG did not show any preferential targeting to the cell cortex (Figures 3E, 3F, upper panels, and S3G). When 480AnkG-GFP was co-expressed with its membrane partner KvNav and TRIM46-mCherry, we observed a clear cortical targeting of AnkG and TRIM46 (Figures 3E, 3F, lower panels, and S3H). TRIM46 alone, which decorates MTs in their proximal parts and localizes to acetylated MTs (Figure S3J) did not localize close to the plasma membrane (Figure S3I). Live imaging in COS-7 cells revealed that 480AnkG-GFP, co-expressed with TRIM46-mCherry alone, was found along the MT lattice but still tracked dynamic MT plus ends (Figure 3G, upper panel). However, when targeted to the membrane by co-expressing KvNav, 480AnkG-GFP colocalized with TRIM46-mCherry bundles and was distributed along the MT lattice (Figure 3G, lower panel) confirming that in these conditions 480AnkG could recruit TRIM46-positive MT bundles to plasma membrane. Interestingly, membrane-attached 480AnkG-GFP efficiently recruited TRIM46-mCherry-positive MT bundles (Figures 3H and 3J) but was not able to target PRC1-positive bundles to the membrane (Figures 3I and 3J).

Neither 270AnkG-GFP nor 480AnkG-NN-GFP were able to localize to TRIM46-induced MT bundles (Figures S3L and S3M), suggesting that EB-binding was required for 480AnkG to be recruited to TRIM46-bound MT lattices. The 480AnkGtail construct also failed to accumulate along the TRIM46-decorated MT lattices and remained enriched at MT plus-ends (Figures S3L and S3M), suggesting that in addition to binding to EB proteins, also the 480AnkG N terminus is important for binding to MTs bundled by TRIM46. To validate this idea, we engineered a 270AnkG isoform able to track MT plus-ends, 270AnkG+TIP, by adding one SxIP motif in its C terminus. The 270AnkG+TIP efficiently accumulated at MT plus-end where it colocalized with EB1 (Figures S3L and S3N). Like 480AnkG, 270AnkG+TIP but not 270AnkG showed strong overlap with TRIM46-decorated MTs (Figures S3L and S3N). Together, these results indicate that 480AnkG specifically recruits TRIM46-bundled MTs to the plasma membrane in an EB-dependent manner.

### TRIM46 Orients and Stabilizes MTs Recruited at the Membrane by AnkG

**Figure 4:**
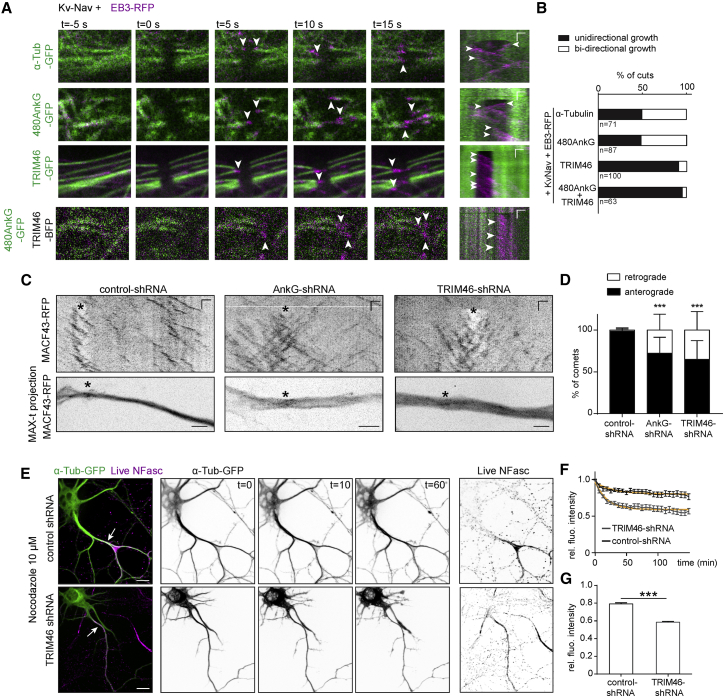
TRIM46 Orients and Stabilizes MTs in the AIS (A and B) Photo-ablation of MTs labeled with indicated constructs in COS-7 co-expressing Kv-Nav and EB3-RFP (A). Kymographs (B) are shown on the right. Scale bars show 1 μm (horizontal) and 15 s (vertical). Regrowth events are indicated by arrowheads. The percentage of cuts is shown in (E). n is the number of cuts, in N ≥ 10 cells. (C and D) Photo-ablation of MTs in the proximal axon of DIV8 neurons co-transfected at DIV0 with indicated constructs (C). Lower panels show the maximum-time projections of MACF43-RFP imaging. Asterisks show the ablation sites. Scales are 1 μm (horizontal) and 15 s (vertical). The mean percentage of comets after ablation is shown in (D). 2-way ANOVA, p = 0.0004 for AnkG-shRNA, p < 0.0001 for TRIM46-shRNA compared to control-shRNA. n = 10 neurons, N = 2. (E–G) DIV4 hippocampal neurons transfected at DIV1 with indicated construct and imaged after addition of 10 μM Nocodazole. (E) shows still frames at 0, 10, and 60 min. (F) Fluorescence intensity of α-tub-GFP in the proximal axon of control (n = 20, black) or TRIM46 knockdown neurons (n = 24, gray) after addition of Nocodazole and normalized to the first frame. Fitted one-phase decay curves are shown in orange. (G) shows the plateau values obtained from the fits, unpaired t test, p < 0.0001. Scale bars are 2 μm in (C) and 10 μm in (E). See also Figure S4.

To test the orientation of TRIM46-labeled MTs recruited to the plasma membrane, we used a laser-severing assay to record the direction of MT plus-end regrowth (Yau et al., 2014). Upon severing of MTs labeled with tubulin-GFP or 480AnkG-GFP in EB3-RFP and Kv-Nav co-expressing cells, we did not observe any preferential MT orientation (Figures 4A and 4B). However, in TRIM46-GFP-labeled MT bundles, plus-end regrowth after laser severing occurred in a single direction in ∼90% of the cases. The binding of TRIM46 along MT bundles recruited at the plasma membrane by 480AnkG also conferred a uniform MT orientation (Figures 4A and 4B). This result shows that TRIM46 decoration confers a uniform orientation to MT fascicles recruited at the membrane by 480AnkG.

TRIM46 and AnkG were shown to be important for plus-end out MT organization in axons (Van Beuningen et al., 2015; Fréal et al., 2016). In order to assess the orientation of both stable and dynamic polymerizing MTs in proximal axons, we performed laser severing experiments and monitored MT plus-end regrowth by imaging a MT plus end marker MACF43-RFP (Honnappa et al., 2009) in DIV8 neurons co-transfected at DIV0 with control- (Figure 4C, left panel), AnkG- (middle panel), or TRIM46-shRNA (right panel). Control neurons contained ∼100% of plus-end out MTs, whereas AnkG- and TRIM46-depleted neurons showed a significant increase in minus-end out MTs (Figure 4D), further strengthening the importance of AnkG and TRIM46 in organizing the axonal MT network.

We next determined whether TRIM46 can protect MT bundles against nocodazole-induced depolymerization. We imaged live cells expressing NF186-RFP and 480AnkG-GFP with BFP or TRIM46-BFP and treated them with nocodazole or DMSO as a control (Figures S4A–S4C). 480AnkG and NF186 were accumulated at the plasma membrane in control cells; however, they rapidly disappeared after the addition of nocodazole. In contrast, co-expression of TRIM46-BFP preserved 480AnkG/NF186 membrane stretches in nocodazole-treated cells (Figures S4B and S4C). To determine whether the nocodazole-resistant 480AnkG/NF186 membrane structures induced by TRIM46 were associated with MTs, we fixed the cells and stained for tubulin (Figures S4E and S4F). In the absence of TRIM46, no remaining MTs were observed after the addition of the drug. Only when TRIM46-BFP was co-expressed, we detected nocodazole-resistant MTs (Figures S4E and S4F). 480AnkG-GFP, NF186-RFP, or 480AnkG-NN-GFP alone, as well as 480AnkG-GFP in combination with NF186-RFP, were not able to confer nocodazole resistance to MTs (Figure S4G). These data suggest that TRIM46-decorated MT bundles recruited to plasma membrane by 480AnkG are protected against depolymerization. Additionally, we observed that TRIM46 bundling increased the clustering of 480AnkG/NF186 at the plasma membrane (Figures S4B and S4D). Interestingly, when 480AnkG-GFP behaved as a +TIP and as well as when it located along MT lattice upon targeting to the membrane by KvNav, it was preferentially associated with tyrosinated MTs and did not colocalize to acetylated MTs (Figure S4H). When TRIM46-BFP was co-expressed together with 480AnkG-GFP, with or without KvNav, AnkG was associated with bundles that consisted of long-lived, acetylated MTs at their proximal end and tyrosinated MTs at the distal end (Figure S4I).

To investigate whether TRIM46 also protects axonal MTs against depolymerization, we expressed α-tubulin-GFP together with control- or TRIM46-shRNA and performed time-lapse imaging before and after nocodazole addition (Figures 4E–4G). Quantification revealed that the tubulin intensity in the proximal axon markedly decreased in neurons depleted of TRIM46 in the presence of nocodazole (Figures 4E–4G). These results show that TRIM46 stabilizes axonal MTs.

### TRIM46 Generates Long and Stable MT Bundles *In Vitro* by Promoting Rescues

**Figure 5:**
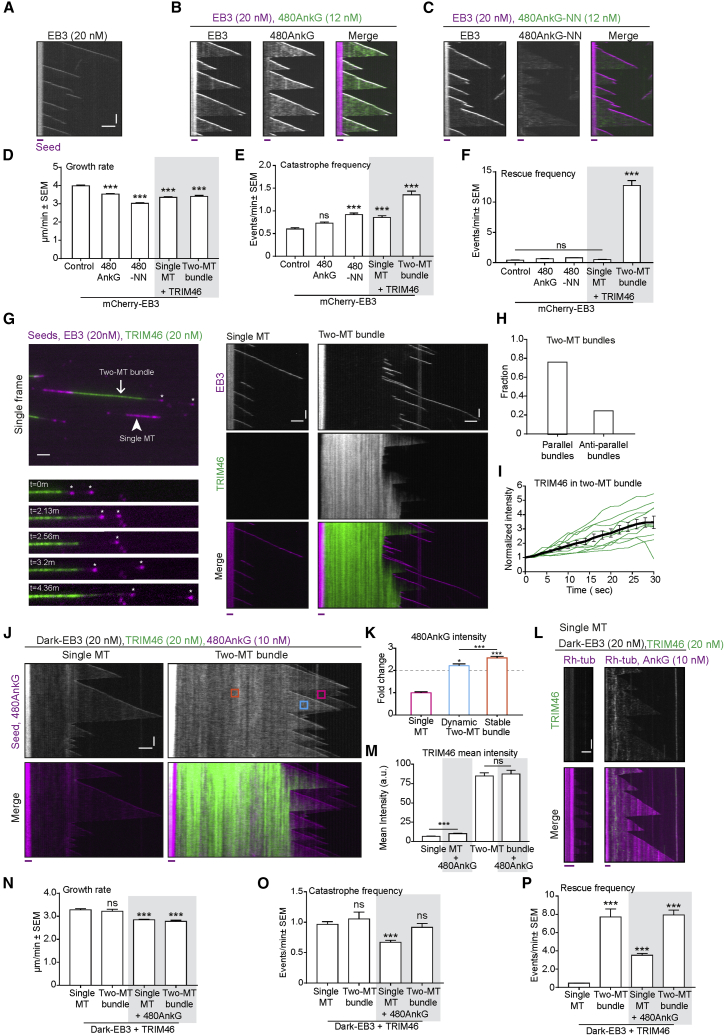
TRIM46 Promotes Rescues within Parallel MT Bundles and Shows Weak Interaction with 480AnkG *In Vitro* (A–C) Kymographs showing MTs dynamics grown in the presence of mCherry-EB3 alone (A) or together with 480AnkG-GFP (B) or 480AnkG-NN-GFP (C). (D–F) Quantification of MT plus-end growth rate (D), catastrophe frequency (E) and rescue frequency (F) in the presence of mCherry-EB3 (n = 213) together with 480AnkG-GFP (n = 388), 480AnkG-NN-GFP (n = 458), or GFP-TRIM46 (n = 235 for single MTs and n = 91 for two-MT bundles), 2–3 assays per condition. One-way ANOVA with Tukey’s multiple comparisons test, ns p > 0.05, ^∗∗∗^p < 0.001. (G–I) (G) Left panels: single frame (top) of a time-lapse video showing single MT (arrowhead) and two-MT bundle (arrow) growing from rhodamine-tubulin-labeled GMPCPP seeds in the presence of mCherry-EB3 and GFP-TRIM46 and still pictures (bottom) at indicated time points (in min). Corresponding kymographs are shown in the middle panel. (H) Fraction of parallel and anti-parallel TRIM46-decorated two-MT bundles (n = 41 bundles). (I) Average intensity (±SEM, black) of GFP-TRIM46 over time in two-MT bundles, normalized to its intensity at the time when bundles form (n = 10 events in green, from 7 TRIM46-positive two-MT bundles, three assays per condition). (J and K) Kymographs (J) showing 480AnkG-mCherry dynamics of single MTs or TRIM46 MT bundles in the presence of dark-EB3. 480AnkG-mCherry mean intensity (K) on single MTs (magenta) or two-MT TRIM46-bundles (dynamic, cyan; stable, orange) normalized to average mean intensity on single MTs, from 1 μm^2^ 33–76 regions of interest (ROIs) of 7–15 TRIM46-decorated two-MT bundles from 6–8 assays. Error bars show 95% confidence interval. One sample t test was carried out to test whether fold change in AnkG intensity on TRIM46-bundles is more than 2, ^∗^p < 0.05, ^∗∗∗^p < 0.001. One-way ANOVA was used to test whether the change in 480AnkG intensity was different in dynamic compared to stable two-MT bundle, ^∗∗^p = 0.0050. (L) Kymographs illustrating GFP-TRIM46 fluorescence intensity on single MTs grown in the presence of dark-EB3, 14.5 μM porcine tubulin, and 0.5 μM rhodamine-tubulin without (left) or with 480AnkG-mCherry (right). (M) Mean intensity of GFP-TRIM46 on single MTs or TRIM46- bundles with or without 480AnkG-mCherry, from 1 μm^2^ 80–140 ROIs from 8–14 single MTs or TRIM46 two-MT bundles, 3 assays per condition. Error bars are SEM. Two-tailed unpaired t test, ns p > 0.05, ^∗∗∗^p < 0.001. (N–P) Quantification of MT plus-end growth rate (N), catastrophe frequency (O), and rescue frequency (P) in the presence of dark-EB3 and GFP-TRIM46 together with 480AnkG-mCherry and 15 μM porcine tubulin or 14.5 μM porcine tubulin and 0.5 μM rhodamine-tubulin. n = 139 and n = 188 single MTs without or with 480AnkG, respectively, and n = 26 and n = 87 TRIM46-decorated MT bundles without or with 480AnkG, respectively, 2–3 assays per condition. One-way ANOVA, ns p > 0.05, ^∗∗∗^p < 0.001. Scale bars in all the kymographs represent 2 μm (horizontal) and 60 s (vertical). The red line below each kymograph represents rhodamine-labeled GMPCPP-stabilized MT seeds. See also Figure S5 and Table S1.

In order to test whether TRIM46 can autonomously confer uniform orientation and stability to 480AnkG-decorated MT bundles, we performed *in vitro* MT dynamics reconstitution assays as described previously (Bieling et al., 2007, Mohan et al., 2013, Sharma et al., 2016), using purified 480AnkG and TRIM46 (Figures S5A and S5B). Since 480AnkG interacts directly with EBs (Fréal et al., 2016), we first examined whether it regulates MT plus-end dynamics *in vitro*. We polymerized MTs from GMPCPP-stabilized seeds in the presence of EB3 alone (Figure 5A), together with 480AnkG (Figure 5B) or with 480AnkG-NN (Figure 5C) and recorded their dynamics using total internal reflection fluorescence (TIRF) microscopy. 480AnkG tracked the plus ends of growing MTs and also displayed some decoration of MT lattice (Figure 5B). The 480AnkG-NN mutant that does not bind to EBs, and the wild-type 480AnkG in the absence of EBs failed to track MT plus-ends and showed only weak MT lattice binding (Figures 5C and S5E). This was consistent with the findings reported previously in COS-7 cells and rat hippocampal neurons (Fréal et al., 2016; Figures 1E and 1F). *In vitro*, 480AnkG did not have any strong impact on MT dynamics (Figures 5D–5F): it mildly reduced MT growth rate but did not significantly affect catastrophe or rescue frequencies (Figures 5D–5F).

Next, we reconstituted MT dynamics in the presence of TRIM46 (Figure 5G). TRIM46 did not bind to single MTs on its own (Figure 5G, arrowhead and middle panel; Video S2) but got recruited to MT bundles (Figure 5G, arrow and right panel). Consistently with previous findings in cells (van Beuningen et al., 2015), TRIM46 preferentially bundled MTs in a parallel manner (Figure 5H). TRIM46 was abundantly present on the overlapping MTs and showed gradual enrichment on the fresh MT overlaps generated by MT growth (Figure 5I). These data indicate that TRIM46 is an autonomous parallel MT bundling factor, which requires presence of at least two closely apposed MTs for an efficient MT interaction.

Video S2. TRIM46 Bundles Parallel MTs and Promotes Rescues of MTs within the Bundle, Related to Figure 5 Time-lapse movie of an *in vitro* reconstitution experiment showing a TRIM46 decorated two-MT bundle (arrow) and a single MT (arrowhead) growing from rhodamine-tubulin-labeled GMPCPP seeds in the presence of 15 μM tubulin, 20 nM mCherry-EB3 and 20 nM GFP-TRIM46. The movie consists of 301 frames recorded with 2 s interval between frames and 100 ms exposure time. Scale bar represents 5 μm.

Like 480AnkG, TRIM46 had little effect on the growth rate of individual MTs (Figure 5D), but, once accumulated along a MT bundle, it potently promoted rescues of MTs within the bundle (Figures 5F and 5G, bottom-left panel), thus leading to their progressive elongation and stabilization. The ability of TRIM46 to prevent MT depolymerization within parallel bundles explains why MTs still persist in COS-7 cells, when they are treated with nocodazole, which promotes MT disassembly by sequestering soluble tubulin (Figures S4A and S4B). We also observed a mild increase in catastrophe frequency within TRIM46-decorated MT bundles (Figure 5E), and overall MT plus ends within TRIM46-decorated MT bundles remained highly dynamic. The fact that the distal part of such bundles remains dynamic and can accumulate EBs and 480AnkG might explain the partial spatial separation of TRIM46 and 480AnkG on bundled MTs (Figures 3B and 3C).

We next tested the potential interplay between 480AnkG and TRIM46. When MTs were grown in the presence of both 480AnkG and TRIM46 (Figure 5J), 480AnkG was slightly enriched along the TRIM46-decorated MT shafts in two-MT bundles (Figure 5K). This enrichment was more pronounced on the stable (orange box in Figure 5J) as compared to dynamic (blue box in Figure 5J) lattices within the bundles. Since stable MT parts had higher accumulation of TRIM46 than the freshly grown MT lattices within the bundle (Figure 5I), this suggested that 480AnkG enrichment was indeed dependent on TRIM46 and not on the presence of two closely apposed MTs. To test this idea further, we performed *in vitro* experiments with 480AnkG and another MT bundling factor, PRC1 (Loïodice et al., 2005, Mollinari et al., 2002). As previously described, we observed that PRC1 did not bind to single MTs, but decorated MT bundles (Bieling et al., 2010, Subramanian et al., 2013). In contrast to what we observed with TRIM46, we found that compared to single MTs, the intensity of 480AnkG along two-MT PRC1 bundles was exactly twice as high on freshly formed PRC1 bundles and even slightly lower on older bundles that were densely populated by PRC1 (Figures S5C and S5D). This suggested that mere bundling is not sufficient to cause enrichment of 480AnkG on MTs but rather the presence of TRIM46 is required for recruitment of 480AnkG to MT bundles. To support the specificity of 480AnkG enrichment along TRIM46-decorated MT bundles, we reconstituted MT dynamics in the presence of TRIM46 and 480AnkG or EB-binding-deficient 480AnkG-NN, without adding EBs in the assay. As expected, we observed very weak lattice binding of AnkG on single MTs in the absence of EBs (Figures S5E–S5G). However, TRIM46 still weakly but significantly recruited 480AnkG to MT bundles even in the absence of EBs and promoted a similar enrichment of the EB-binding-deficient mutant 480AnkG-NN on MT bundles (Figures S5F–S5I). Furthermore, in the presence of 480AnkG, TRIM46 was weakly bound to single MTs (Figures 5L and 5M), whereas its accumulation on two-MT bundles remained unaltered (Figure 5M). Moreover, in the presence of 480AnkG, TRIM46 was able to weakly promote rescues even on single MTs (Figure 5P), while the effects on MT growth rate and catastrophe frequency were mild (Figures 5N and 5O). Our results show that TRIM46 is a potent rescue factor that triggers some enrichment of 480AnkG along the MT lattice. Interestingly, 480AnkG also mildly increases the affinity of TRIM46 for single MTs further supporting our findings about the weak MT-dependent cooperativity between 480AnkG and TRIM46. Taken together, our data demonstrate that TRIM46 can autonomously generate long and stable parallel MT arrays, which remain dynamic at their distal parts. These arrays concentrate EBs and Ankyrin-G, which in turn provide connections to the plasma membrane.

### TRIM46 Promotes Efficient Trafficking of NF186 Vesicles to the Proximal Axon

**Figure 6:**
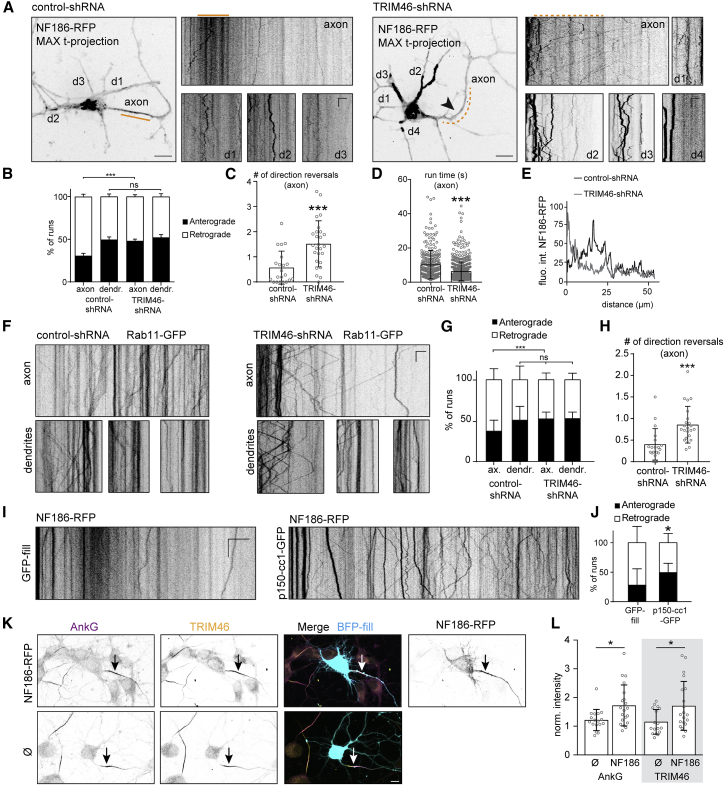
TRIM46 Promotes Retrograde Transport of NF186 to the Proximal Axon (A) Maximum time projection from time-lapse imaging of DIV4 neurons transfected at DIV0 with indicated constructs. Kymographs of axon and dendrites are shown. Orange lines highlight the proximal axon. Scale is 5 μm (horizontal) and 15 s (vertical). (B) Percentage of NF186-RFP runs in DIV4 neurons co-transfected at DIV0 with indicated constructs. n = 20 neurons, N = 2, 2-way ANOVA, ^∗∗∗^p = 0.0002, ns: p = 0.916. (C and D) Quantification of NF186-RFP vesicle dynamics in DIV4 neurons co-transfected at DIV0 with indicated constructs. Mann-Whitney in (C), p = 0.0003, n = 21–26 axons, and in (D), p < 0.0001, n = 279–645 runs. (E) Fluorescence intensity profiles of NF186-RFP along the axons of neurons shown in (A). (F–H) Kymographs (F) of Rab11-GFP vesicles in DIV3 neurons co-transfected at DIV0 with indicated constructs. Scale is 5 μm (horizontal) and 3 s (vertical). (G) Percentage of runs. (H) Mean number of direction reversals per axon. n = 18 neurons, N = 2. In (G), two-way ANOVA, ns p = 0.5, ^∗∗∗^p < 0.001. In (H), Mann-Whitney test, ^∗∗∗^p = 0.00012. (I and J) Kymographs (I) of NF186-RFP in the axons of DIV3 neurons co-transfected at DIV0 with indicated constructs. Scale is 10 μm (horizontal) and 5 s (vertical). (J) Percentage of runs. Two-way ANOVA, ^∗^p = 0.0104, n = 20 neurons, N = 2. (K and L) DIV5 hippocampal neurons (K) co-transfected at DIV1 with indicated constructs and stained for AnkG and TRIM46. (L) Intensity of AnkG and TRIM46 staining at the AIS normalized to neighboring non-transfected neurons. One-way ANOVA with Holm-Sidak’s multiple comparison test, p = 0.037 for AnkG, p = 0.020 for TRIM46. n = 17 neurons, N = 2. Scale bars are 10 μm in (A) and (K). See also Figure S6.

We have shown that 480AnkG mediates the membrane recruitment of TRIM46-decorated MT bundles and that TRIM46 is a rescue factor that protects against MT depolymerization. We hypothesize that this unique membrane-MT organization may allow for efficient targeting of the AIS membrane proteins to the proximal axon. Using time-lapse imaging of DIV4 neurons expressing control shRNA, we observed mobile NF186-RFP vesicles trafficking in the axon, as well as an immobile pool of NF186 in the proximal axon (Figures 6A and 6E). NF186 vesicles colocalized with endosomal markers, such as Rab5-GFP and Rab11-GFP as well as endogenous Rab11, in fixed (Figure S6A) and live neurons (Figures S6B and S6C). In neurons depleted of TRIM46, we observed significant changes in NF186-RFP vesicle mobility in the axon and a frequent switch in the transport direction (Figures 6B–6E). In control axons (within the first ∼100 μm), NF186-RFP vesicles were mainly transported back to the cell body (∼70% retrograde, Figure 6B), whereas in the axons of TRIM46-depleted neurons, NF186 vesicles were equally transported back and forward (∼50% retrograde, Figure 6B). This change in transport direction in TRIM46-depleted axons was accompanied by a strong increase in the number of reversals (∼3-fold change, Figures 6C and S6D) and a decrease in the mean run time (Figures 6D and S6F). Consistently, we verified that Rab11 transport is affected in TRIM46-depleted neurons. We transfected DIV0 neurons with Rab11-GFP in combination with the control or TRIM46-shRNA (Figure 6F). We observed changes in the directionality of runs and in the number of reversals. In the axons of DIV3 control neurons, retrogradely moving Rab11 vesicles were more abundant than anterograde ones (Figure 6G). In contrast, in axons of neurons depleted of TRIM46, Rab11 vesicles moved in both directions with the same frequency, as it was the case in dendrites, and switched direction more often (Figures 6G and 6H).

The bias toward retrograde transport of NF186 in the axon suggested a role for the minus-end directed motor dynein. We analyzed NF186-RFP trafficking in the axons of neurons expressing GFP or p150cc1-GFP, a dominant-negative form of the dynactin adaptor p150, which is reported to perturb dynein function (Kuijpers et al., 2016). As reported for TRIM46 knockdown, perturbing dynein function induced changes in NF186 vesicles transport, with a significant increase in the fraction of anterograde runs compared to control (Figure 6J). These results indicate that TRIM46-mediated plus-end out MT orientation allows for the efficient retrograde transport of NF186 vesicles from the distal to the proximal axon via the endosomal pathway in a dynein-dependent manner.

Controlled trafficking and clustering of AIS membrane proteins, adhesion molecules, and ion channels is crucial for neuronal activity (Kole et al., 2008), and it was shown to contribute to AIS formation and maintenance by influencing AnkG accumulation in this region (Alpizar et al., 2019, Leterrier et al., 2017, Xu and Shrager, 2005, Zonta et al., 2011). We co-expressed NF186-RFP (Figure 6K) or an empty vector together with a BFP-fill in DIV1 neurons and stained for endogenous AnkG and TRIM46 at DIV5 (Figure 6K). We observed that the overexpression of both NF186 or KvNav (Figure S6G) increased the accumulation of endogenous AnkG and TRIM46 at the AIS (Figures 6L and S6H). We conclude that a membrane partner can promote clustering of both AnkG and TRIM46 at the AIS in developing neurons. Other AIS membrane proteins, such as Kv7 or NF186, as well as AnkG modifications such as palmitoylation, could possibly have an effect on AnkG/TRIM46 accumulation at the AIS.

### AnkG Inhibits NF186 Endocytosis Resulting In Stable Accumulation at the AIS

**Figure 7:**
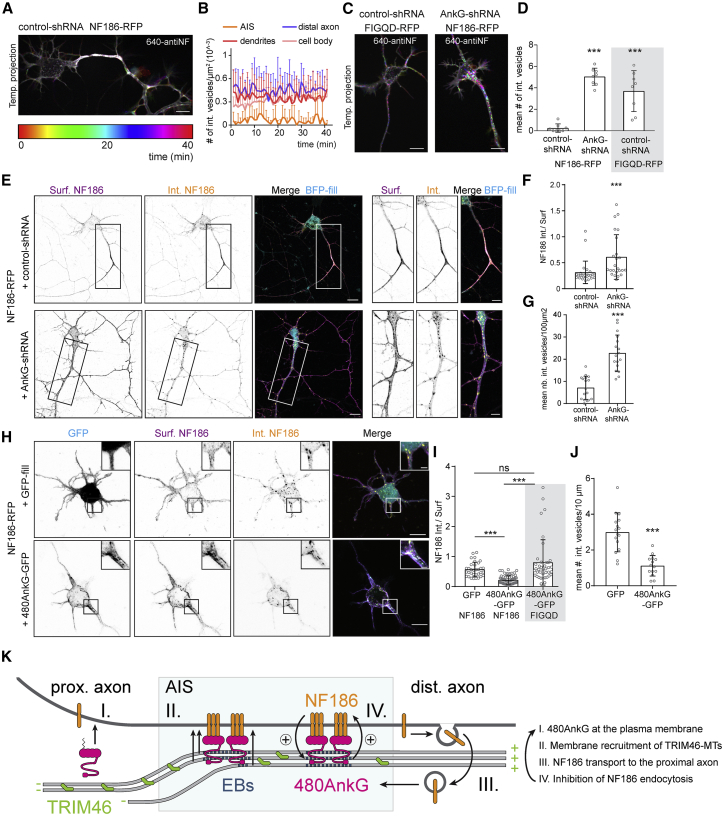
AnkG Allows for the Stable Accumulation of NF186 at the AIS by Inhibiting Its Endocytosis (A and B) Temporal-color coded maximum projection (A) from live imaging of a DIV4 neuron co-transfected at DIV0 with indicated constructs and incubated with a fluorescently tagged anti-NF antibody (640-antiNF). (B) Number of internalized NF vesicles in indicated compartments. (C and D) Temporal-color coded maximum projections (C) from live imaging of DIV4 neurons co-transfected at DIV0 with indicated constructs incubated with 640-antiNF. (D) Mean number of 640-antiNF vesicles during the first 5 min post incubation in the proximal axons. One-way ANOVA, ^∗∗∗^p < 0.0001, n = 8 neurons, N = 2. (E–G) DIV4 neurons (E) co-transfected at DIV0 with indicated constructs. (F) Fluorescence intensity ratio of internalized over surface NF186 in the proximal axon. (G) Mean number of internalized NF186 vesicles per 100 μm^2^ of axon. Mann-Whitney test, p = 1.7^∗^10^–7^ n = 17 axons. (H–J) DIV1 neurons (H) transfected at DIV0 with indicated constructs. (I) Fluorescence intensity ratio of internalized over surface NF186 in the first 10 μm of proximal neurites. Kruskal-Wallis test, Dunn’s multiple comparison test. ns, p > 0.99, ^∗∗∗^p < 0.0001, n = 33 ROIs. (J) Number of internalized NF186 vesicles per 10 μm. Unpaired t test, ^∗∗∗^p < 0.0001, n = 14 neurons, N = 2. (K) Model of the molecular pathways involved in AIS formation. See text for details. In (A), (C), and (D), scale bars are 10 μm; in (E), scale bars are 10 and 2 μm in the zooms; and in (H), scale bars are 10 and 5 μm in the zooms. See also Figure S7.

We next speculated that the scaffold formed by AnkG may allow for local accumulation of the AIS membrane proteins in the proximal axon by selectively preventing their endocytosis at the AIS. To track membrane NF186 in live neurons, we performed an antibody uptake experiment using a fluorescently labeled antibody against NF186 on DIV4 neurons expressing NF186-RFP (Figures 7A–7D). In the first minutes after antibody incubation, high numbers of internalized NF186 vesicles were detected in dendrites, cell body, and distal axons (>50 μm away from the soma), whereas the AIS contained a lower number of vesicles (Figures 7A and 7B). The number of internalized-NF186 vesicles in the proximal axon increased dramatically upon depletion of AnkG (Figures 7C, left panel, and 7D) or when a NF186 mutant incapable of binding to AnkG was expressed (wt-FIGQY to mutant-FIGQD; Boiko et al., 2007, Zhang et al., 1998; Figures 7C, right panel, 7D). In order to measure the endocytosis rate, we quantified the ratio of internalized-to-surface NF186 in DIV4 neurons expressing control- or AnkG-shRNA (Figures 7E–7G) and observed a robust increase in the number of NF186-positive vesicles and an enhanced endocytosis rate in neurons depleted of AnkG (Figures 7F and 7G). To question the direct role of AnkG in opposing endocytosis of NF186, we transfected DIV0 neurons, not yet expressing endogenous AnkG, with NF186-RFP together with 480AnkG-GFP or with GFP (Figures 7H–7J). In 480AnkG-GFP-expressing neurons, the surface pool of NF186 colocalized with 480AnkG-GFP along neurites (Figure 7I, lower panel and zoom), and the internalized signal was markedly decreased (Figures 7I and 7J), suggesting that 480AnkG prevents NF186 endocytosis. Next, we used the NF186-FIGQD mutant to test whether the direct interaction between AnkG and NF186 is responsible for the lower endocytosis rate. NF186-FIGQD did not accumulate in the proximal axon of DIV3 neurons and showed no colocalization with 480AnkG-GFP (Figures S7A and S7B). In DIV1 neurons, NF186-FIGQD co-expressed with 480AnkG-GFP was taken up at a rate similar to that observed for the wild-type NF186 in the absence of 480AnkG-GFP (Figures 7I and S7C). Our results indicate that AnkG, by directly interacting with NF186, is able to prevent NF186 endocytosis allowing for stable accumulation at the AIS.

## Discussion

### Role of AnkG in MT Organization at the AIS Membrane

The crucial role of AnkG in AIS formation and maintenance has been extensively demonstrated *in vitro* and *in vivo* (Hedstrom et al., 2008, Sobotzik et al., 2009). More recently, the 480-kDa isoform has emerged as the essential player in this process; however, the mechanisms used by this giant scaffold protein are far from being understood (Fréal et al., 2016, Jenkins et al., 2015b). Recent studies indicate that AnkG’s role in AIS assembly requires both its membrane targeting and its association with MTs via EBs, but the functional pathways implicated have remained speculative (Fréal et al., 2016, He et al., 2012, Leterrier et al., 2011, Leterrier et al., 2017). We here describe novel functions for 480AnkG underpinning its pivotal role in AIS formation and maintenance (Figure 7K). First, we show that 480AnkG targets and anchors MTs in the vicinity of the plasma membrane. This newly described function of 480AnkG depends both on its membrane association and its binding to EBs, since AnkG truncations lacking one of these properties lose the ability to organize MTs. The direct control of MT organization by membrane-480AnkG was never reported and is likely to explain the axonal MT alterations described in AnkG-deficient neurons *in vitro* and *in vivo* (Fréal et al., 2016, Hedstrom et al., 2008, Leterrier et al., 2011, Sobotzik et al., 2009). Next to the direct interaction with Nav or NF186, other AIS channels such as Kv7 (Pan et al., 2006, Rasmussen et al., 2007) or the adhesion molecule NrCAM (Dzhashiashvili et al., 2007, Hedstrom et al., 2007) possibly mediate AnkG recruitment at the plasma membrane. Palmitoylation of the Cys70 residue of AnkG was reported to be crucial for its role in driving AIS assembly (He et al., 2012), and it would be interesting to test whether palmitoylation of 480AnkG isoform in neurons is required for AIS formation.

Interestingly, the membrane recruitment of MTs induced by 480AnkG in COS-7 cells is consistent with super-resolution microscopy-based observations showing that MTs in the AIS are mostly found within ∼80 nm of the plasma membrane (Leterrier et al., 2015). We propose that the local membrane-MT coupling by 480AnkG could provide specific properties to this specialized axonal compartment. Since AnkG is integrated into the periodic axonal spectrin-actin rings, the localization of AnkG at the crosspoints between transversal actin rings and longitudinal MTs running under the plasma membrane may create a “grid-like” organization coupling actin and MT networks. This cytoskeleton organization may allow for cooperative control of axonal diameter and homeostatic plasticity (Berger et al., 2018, Grubb et al., 2011). Additionally, the integrated AIS-MT organization may control axonal cargo trafficking by bringing regulatory proteins anchored at the AIS membrane within close range of the motor-cargo complexes, explaining why axonal carriers proceed and somatodendritic cargos stop and reverse. For instance, dynein regulatory protein Ndel1 localizes to the AIS by binding to AnkG and stimulates retrograde transport of somatodendritic vesicles at the AIS (Kuijpers et al., 2016).

### Mechanisms of Membrane Protein Accumulation in the AIS

Although a lot of attention has recently been given to the role of the proximal axon as a transport checkpoint (Leterrier, 2018), much less is known about the transport mechanism of AIS components themselves into the proximal axon. Despite NF186’s function in maintaining the AIS and recruiting specialized extracellular matrix structures (Alpizar et al., 2019, Hedstrom et al., 2007, Zonta et al., 2011), little is known about how this transmembrane protein gets targeted and retained at the AIS. Some papers suggest that clathrin-mediated endocytosis is important for NF186 surface distribution in neurons (Boiko et al., 2007, Yap et al., 2012). In this study, we show that, upon non-specific targeting of NF186 to both the axon and dendrites, NF186 gets endocytosed except at the AIS, where the interaction with AnkG locally blocks its internalization (Figure 7K). The NF186 interaction with AnkG was shown to depend on phosphorylation of its cytoplasmic FIGQY motif (Tuvia et al., 1997, Zhang et al., 1998), suggesting an additional regulatory mechanism, in which phospho-NF186 does not associate with AnkG and therefore gets internalized at the AIS. It is possible that AnkG may also inhibit the endocytosis of other transmembrane proteins such as Nav at the AIS. After a non-targeted delivery to all compartments and removal by endocytosis outside the AIS (Fache et al., 2004), Nav anchoring relies on direct interaction with AnkG, which is regulated by a phosphorylation-dependent mechanism implicating the kinase CK2 (Bréchet et al., 2008, Garrido et al., 2003, Lemaillet et al., 2003). A function for AnkG in organizing membrane micro-domains by locally inhibiting endocytosis was recently reported for somatodendritic GABAergic synapses maintenance and lateral membrane assembly in MCDK cells (Cadwell et al., 2016, Jenkins et al., 2015a, Jenkins et al., 2013, Tseng et al., 2015). This newly described function of AnkG, wherein AnkG hinders the endocytosis of membrane proteins in a potentially phospho-dependent manner opens new ideas around the pathways implicated in AIS homeostasis and plasticity (Berger et al., 2018, Evans et al., 2013, Grubb et al., 2011). Once internalized by endocytosis, NF186-containing endocytic vesicles are subsequently retrieved from the distal axon and travel back to the proximal axon in a dynein-dependent manner. The polarized distribution of somatodendritic cargos such as α5-integrin or transferrin and glutamate receptors was also shown to rely on axonal retrieval by Rab5- and Rab11-positive vesicles (Guo et al., 2016, Koseki et al., 2017). Interestingly, we observed that, in the absence of TRIM46, retrograde transport of NF186 as well as Rab11 is markedly perturbed. Impaired axonal retrieval of somatodendritic and AIS proteins could be responsible for the polarity defects observed in TRIM46-depleted neurons (van Beuningen et al., 2015). Although trafficking of the dendritic AMPA receptor subunit GluR2 was unaffected by TRIM46 depletion (van Beuningen et al., 2015), we cannot exclude that the changes in NF186 vesicles transport we report in the absence of TRIM46 could also result from the abnormal entry of somatodendritic motors into the axon. Altogether our data reveal that the formation of a stable and functional AIS relies on the cooperative coupling between directional transport and local membrane protein stabilization.

### Functional Cross-talk between AnkG and TRIM46

Our *in vitro* reconstitution assays for the first time reveal the functional relationship between the MT organizer TRIM46 and the membrane scaffold 480AnkG. Interestingly, we observed that TRIM46 does not bind to single MTs but to the lattice of at least two closely apposed parallel MTs. We propose that the 480AnkG-induced MT fascicles running along the plasma membrane facilitate TRIM46 binding along the MT lattice. This would explain the strong effects of AnkG knockdown on TRIM46, where ∼90% of the neurons lacking AnkG showed altered TRIM46 accumulation in the proximal axon. We show that TRIM46 binding to parallel MTs induces their stabilization by strongly increasing rescue frequency. Importantly, the accumulation of TRIM46 on bundled MTs occurs with a time delay, and as a result MT plus ends within the bundles remain dynamic and can accumulate EBs and 480AnkG. Furthermore, 480AnkG and TRIM46 display a weak cooperativity: TRIM46 mildly stimulates recruitment of 480AnkG to the MT lattice and 480AnkG mildly increases the affinity of TRIM46 for single MTs. These weak interactions promote formation of membrane-associated MT fascicles without causing complete convergence of the TRIM46 and 480AnkG-occupied domains. Interestingly, in COS-7 cells we observed that membrane-480AnkG is able to target MTs of mixed orientation to the membrane, the uniform orientation is being caused by TRIM46 co-expression. In TRIM46 knockdown neurons, AnkG shows a reduced accumulation at the AIS but is most likely still able to recruit (some) MTs close to the membrane. Even if it is still present, this MT recruitment at the membrane by AnkG is not sufficient to prevent the transport changes of NF186 and Rab11 vesicles that we report in the axons of neurons lacking TRIM46, revealing different but cooperative functions of TRIM46 and AnkG in the formation of the AIS.

The *in vitro* results, combined with our cellular data, highlight the molecular pathways for AIS formation. The cooperation between TRIM46-mediated MT organization and 480AnkG-mediated membrane scaffolding allows for the directional targeting and local retention of NF186 at the AIS. The stabilization of NF186 at the AIS in turn increases the submembrane concentration of AnkG in the proximal axon. Higher AnkG concentration in turn strengthens the membrane anchoring of MTs and facilitates TRIM46-lattice stabilization and preferential orientation, driving a more efficient and directed retrieval of NF186 to the proximal axon. Since the AIS was shown to be a dynamic compartment (Grubb and Burrone, 2010, Kuba et al., 2010), it would be interesting to investigate whether and how the mechanism of AIS assembly play a role in activity-induced AIS plasticity. The phosphatase calcineurin has been shown to play a role in this phenomenon (Evans et al., 2013), but the target proteins as well as the pathways implicated remain unclear. We here show that AnkG locally controls NF186 endocytosis, in a potentially phosphorylation-dependent manner, which could represent one part of the cascade regulating AIS repositioning during plasticity. Interestingly, next to the role of the scaffolding protein AnkG, interactions with the MAPs DCX and MAP1B have also been reported to participate in the regulation of NF186 and Nav1.6 endocytosis in neurons (Solé et al., 2019, Yap et al., 2012). These data further emphasize the idea of strong cooperation between membrane-associated proteins and the cytoskeleton during AIS assembly and dynamics.

## STAR★Methods

### Key Resources Table

**Table:** 

REAGENT or RESOURCE	SOURCE	IDENTIFIER
**Antibodies**		

rabbit anti-EB2	From A. Akhmanova, Stepanova et al., 2003	N/A
mouse anti-AnkG	Neuromab	Cat# N106/36; RRID: AB_10673030
mouse anti-AnkG, clone N106/20	Neuromab	Cat# N106/20; RRID: AB_2750699
mouse anti-EB1, clone 5/EB1	BD Transduction Laboratories	Cat# 610535; RRID: AB_397892
mouse anti-Rab11, clone47	BD Transduction Laboratories	Cat# 610656; RRID: AB_397983
chicken anti-MAP2	Abcam	Cat# ab5392; RRID: AB_2138153
chicken anti-GFP	Abcam	Cat# ab13970; RRID: AB_300798
rabbit anti-α-tubulin	Abcam	Cat# ab52866; RRID: AB_869989
rat anti-tyrosinated tubulin	Abcam	Cat# ab6160; RRID: AB_305328
rabbit anti-GFP	MBL International	Cat# 598S; RRID: AB_591816
mouse anti-GFP, clone 3E6	Life Technologies	Cat# A-11120; RRID: AB_221568
mouse anti-α-tubulin	Sigma-Aldrich	Cat# T6074; RRID: AB_477582
mouse anti-acetylated tubulin	Sigma-Aldrich	Cat# T7451; RRID: AB_609894
mouse anti-β-tubulin	Sigma-Aldrich	Cat# T5201; RRID: AB_609915
goat anti-mouse Alexa405	Life Technologies	Cat# A31553; RRID: AB_22160
goat anti-chicken Alexa405	Life Technologies	Cat# ab175675; RRID: AB_2810980
goat anti-rabbit Alexa405	Life Technologies	Cat# A31556; RRID: AB_221605
goat anti-mouse Alexa488	Life Technologies	Cat# A11029; RRID: AB_138404
goat anti-chicken Alexa488	Life Technologies	Cat# A11039; RRID: AB_142924
goat anti-rabbit Alexa488	Life Technologies	Cat# A11034; RRID: AB_2576217
goat anti-mouse Alexa594	Life Technologies	Cat# A11032; RRID: AB_141672
goat anti-rabbit Alexa594	Life Technologies	Cat# A11037; RRID: AB_2534095
goat anti-mouse Alexa568	Life Technologies	Cat# A11031; RRID: AB_14469
goat anti-rabbit Alexa568	Life Technologies	Cat# A11036; RRID: AB_143011
goat anti-mouse Alexa647	Life Technologies	Cat# A21236; RRID: AB_141725
goat anti-rabbit Alexa647	Life Technologies	Cat# A21245; RRID: AB_2535813
goat anti-mouse Atto 647N	Sigma Aldrich	Cat# 50185; RRID: AB_1137661
goat anti-rabbit STAR 580	Abberior	Cat# 2-0012-005-8; RRID: AB_2810981
goat anti-mouse STAR RED	Abberior	Cat# 2-0002-011-2; RRID: AB_2810982
anti-Pan-Neurofascin	Neuromab	Cat# A12/18; RRID: AB_2282826
rabbit anti-TRIM46	From C. Hoogenraad, van Beuningen et al., 2015	N/A
rabbit anti-EB3	From A. Akhmanova, Stepanova et al., 2003	N/A
rabbit anti-IgG	Agilent	Cat# Z0412, RRID: AB_2810286

**Chemicals, Peptides, and Recombinant Proteins**		

Vectashield mounting medium	Vectorlabs	Cat# H-1000
Mix-n-Stain CF640R	Biotium	Cat# 92245
Nocodazole	Sigma-Aldrich	Cat# M1404; CAS 31430-18-9
Lipofectamine 2000	Invitrogen	Cat# 1639722
Fugene6	Promega	Cat# E2691

**Critical Commercial Assays**		

Rat Neuron Nucleofector kit	Amaxa	Cat# VVPG-1003

**Deposited Data**		

Raw mass-spectrometry data	This paper	PXD013685

**Experimental Models: Cell Lines**		

Human Bone Osteosarcoma Epithelial (U2OS)	ATCC	CRL-1573
African Green Monkey SV40-transformed kidneyfibroblast (COS-7)	ATCC	CRL-1651
Human embryonic kidney 293 (HEK)	ATCC	HTB-96

**Experimental Models: Organisms/Strains**		

Rat (Wistar)	Janvier	N/A

**Recombinant DNA**		

TRIM46-GFP	van Beuningen et al., 2015	N/A
TRIM36-mCherry	van Beuningen et al., 2015	N/A
PRC1-mCherry	van Beuningen et al., 2015	N/A
480AnkG-GFP	Fréal et al., 2016	N/A
480AnkG-NN-GFP	Fréal et al., 2016	N/A
480AnkGtail-GFP	Fréal et al., 2016	N/A
270AnkG-GFP	Fréal et al., 2016	N/A
myc-Kv-Nav	Bréchet et al., 2008	N/A
Rab5-GFP	Hoogenraad et al., 2010	N/A
Rab11-GFP	Hoogenraad et al., 2010	N/A
Rab6-GFP	Schlager et al., 2010	N/A
NPY-GFP	Schlager et al., 2010	N/A
HA-NF186-mRFP-FKBP	Kuijpers et al., 2016	N/A
EB3-RFP	Stepanova et al., 2003	N/A
mEOS-tubulin	Cloin et al., 2017	N/A
TRIM46-BFP	This paper	N/A
pGW1-BFP	Kapitein et al., 2010	N/A
HA-NF186(FIGQD)-mRFP-FKBP	This paper	N/A
C70A-480AnkG-GFP	This paper	N/A
StrepII-480AnkG-GFP	This paper	N/A
StrepII-480AnkG-mCherry	This paper	N/A
StrepII-480AnkG-NN-GFP	This paper	N/A
StrepII-480AnkG-NN-mCherry	This paper	N/A
pTT5-EGFP-N1	Atherton et al., 2017	N/A
pTT5-mCherry-N1	Atherton et al., 2017	N/A
StrepII-GFP-TRIM46	This paper	N/A
StrepII-GFP-PRC1	This paper	N/A
StrepII-GFP-C1	Atherton et al., 2017	N/A
270AnkG+TIP-GFP	This paper	N/A
MACF18-GFP	Honnappa et al., 2009	N/A
β-tubulin-GFP	kind gift from Dr. P. Schätzle and Dr. K. Jiang	N/A
shRNA AnkyrinG	Hedstrom et al., 2007	N/A
shRNA TRIM46	van Beuningen et al., 2015	N/A
pSUPER-shRNA	Brummelkamp et al., 2002	N/A
TRIM46-mCherry	Van Beuningen et al., 2015	N/A

**Software and Algorithms**		

plugin ComDet	Eugene Katrukha	https://github.com/ekatrukha/ComDet
plugin Pro_Feat_Fit	Christophe Leterrier	https://github.com/cleterrier/Measure_ROIs
Fiji	Schindelin, J. et al., 2012	https://imagej.net/Fiji
GraphPad Prism 8	GraphPad	https://www.graphpad.com/scientific-software/
plugin KymoResliceWide v.0.4	Eugene Katrukha	https://github.com/ekatrukha/KymoResliceWide

### Lead Contact and Materials Availability

Further information and requests for resources and reagents should be directed to and will be fulfilled by the Lead Contact Casper Hoogenraad (c.hoogenraad@uu.nl).

### Experimental Model and Subject Details

#### Animals

All experiments were approved by the DEC Dutch Animal Experiments Committee (Dier Experimenten Commissie), performed in line with institutional guidelines of University Utrecht and were conducted in agreement with Dutch law (*Wet op de Dierproeven*, 1996) and European regulations (Directive 2010/63/EU). Female pregnant Wistar rats were obtained from Janvier and were aged at least 10 weeks at the time of delivery. Upon delivery, rats were kept in a controlled 12 h light-dark cycle with a temperature of 22 ± 1°C and were given unrestricted access to food and water. The animals were housed with companions in transparent Plexiglas cages with wood-chip bedding and paper tissue for nest building and cage enrichment. For hippocampal neuron culture experiments obtained from rat embryos, embryos of both gender at E18 stage of development were used. None of the parameters analyzed in this study are reported to be affected by embryo gender. The animals, pregnant females and embryos have not been involved in previous procedures.

#### Heterologous Cell Culture and Transfection

African Green Monkey SV40-transformed kidney fibroblast (COS-7), Human embryonic kidney 293 (HEK) and Human Bone Osteosarcoma Epithelial (U2OS) cells were from ATCC and cultured in DMEM/Ham’s F10 (45%/45%) supplemented with 10% fetal calf serum and 1% penicillin/streptomycin at 37°C and 5% CO_2_. Cell lines were not authenticated by authors after purchase. The cell lines were routinely checked for mycoplasma contamination using LT07-518 Mycoalert assay (Lonza).

Cells were plated on 18mm glass coverslips and transfected with Fugene6 (Promega) according to manufacturer’s protocol.

#### Primary Neuronal Cultures and Transfection

Primary hippocampal neurons cultures were prepared from embryonic day 18 rat brains (both genders). Cells were plated on coverslips coated with poly-L-lysine (37.5 μg/mL) and laminin (1.25 μg/mL) at a density of 100,000/well. Neurons were cultured in Neurobasal medium (NB) supplemented with 2% B27 (GIBCO), 0.5 mM glutamine (GIBCO), 15.6 μM glutamate (Sigma), and 1% penicillin/streptomycin (GIBCO) at 37°C in 5% CO_2_.

Hippocampal neurons were transfected using Lipofectamine 2000 (Invitrogen). Briefly, DNA (1.8 μg/well, of a 12 wells plate) was mixed with 3.3 μL of Lipofectamine 2000 in 200 μL NB, incubated for 30 min, and then added to the neurons in NB at 37°C in 5% CO_2_ for 45 min. Next, neurons were washed with NB and transferred to their original medium at 37°C in 5% CO_2._

Alternatively, hippocampal neurons (400,000 cells) were nucleofected with 3 μg of DNA using the Amaxa Rat Neuron Nucleofector kit (Lonza) according to the manufacturer’s instructions.

### Method Details

#### DNA and shRNA Constructs

The following constructs were already described: TRIM46-mCherry, TRIM46-GFP, TRIM36-mCherry and PRC1-mCherry (van Beuningen et al., 2015), 480AnkG-GFP, 480AnkG-NN-GFP, 270AnkG-GFP, 480AnkGtail-GFP (Fréal et al., 2016), myc-Kv-Nav (Bréchet et al., 2008), Rab5-GFP and Rab11-GFP (Hoogenraad et al., 2010), Rab6-GFP and NPY-GFP (Schlager et al., 2010), HA-NF186-mRFP-FKBP (Kuijpers et al., 2016), EB3-RFP (Stepanova et al., 2003) and mEOS-tubulin (Cloin et al., 2017).

TRIM46-BFP was obtained by inserting TRIM46 into pGW1-BFP (Kapitein et al., 2010) using AscI/SalI restriction sites.

HA-NF186(FIGQD)-mRFP-FKBP was obtained by overlap extension PCR. NF186(FIGQD) was amplified from HA-NF186-GFP (Kuijpers et al., 2016), and the resulting fragment was inserted in the HindIII/AgeI sites of HA-NF186-mRFP-FKBP (Kuijpers et al., 2016) to replace wt-NF186. C70A-480AnkG-GFP was created by first generating a fragment containing the C70A mutation using overlap extension PCR and using this fragment to replace the region between KpnI and AclI sites in 480AnkG-GFP.

StrepII-480AnkG-GFP and -mCherry as well as StrepII-480AnkG-NN-GFP and -mCherry were obtained by insertion of 480AnkG and 480AnkG into pTT5-EGFP-N1 or pTT5-mCherry-N1 (Atherton et al., 2017) using KpnI/AgeI restriction sites.

StrepII-GFP-TRIM46 and StrepII-GFP-PRC1 were obtained by PCR. TRIM46 and PRC1 were amplified from GFP-TRIM46 and GFP-PRC1 (van Beuningen et al., 2015) and inserted into the BglII/SalI sites of a modified StrepII-GFP-C1 vector. 270AnkG+TIP-GFP was obtained by replacing GFP from 270AnkG-GFP by MACF18-GFP (Honnappa et al., 2009) using AgeI/NotI restriction sites. β-tubulin-GFP was a kind gift from Dr. P. Schätzle and Dr. K. Jiang.

Previously described sequences for AnkG-shRNA (Hedstrom et al., 2007) and TRIM46-shRNA (van Beuningen et al., 2015) were cloned into pSUPER (Brummelkamp et al., 2002). Empty-pSUPER was used as a control-shRNA.

#### Antibodies and Reagents

Rabbit anti-TRIM46 was previously described (van Beuningen et al., 2015) as well as rabbit anti-EB3 and rat anti-EB2 (Stepanova et al., 2003).

Mouse anti-AnkG (clone N106/36 and clone N106/20) and anti-Pan-Neurofascin (cloneA12/18) were from Neuromab.

Mouse anti-EB1 (clone 5/EB1), and mouse anti-Rab11 (clone 47/Rab11) were from BD Transduction Laboratories. Chicken anti-MAP2 (ab5392) and anti-GFP (ab13970), rabbit anti-α-tubulin (ab52866) and rat anti-tyrosinated tubulin (ab6160) were from Abcam. Rabbit anti-GFP (598) was from MBL, and mouse anti-GFP (clone 3E6, A-11120) was from Life Technologies. Mouse anti-α-tubulin (B-5-1-2), anti-acetylated tubulin (6-11B-1, T7451) and anti- β-tubulin (T5201) were from Sigma-Aldrich. Corresponding secondary antibodies Alexa-conjugated 350, 405, 488, 568, 594 or 647 goat anti-mouse, anti-rabbit or anti-chicken were used (Life Technologies). Atto 647N Phalloidin was from Atto-Tec.

#### Pharmacological Treatments

Nocodazole (Sigma) was used at 10 μM and DMSO (0.001%) was used as a control.

#### Immunostaining

For immunocytochemistry, cells were fixed for 10 min with warm paraformaldehyde (4%)-sucrose (4%) or for 5 min with methanol (100%) containing 1 mM EGTA at −20°C followed by 5 min paraformaldehyde (4%). Primary antibodies were incubated overnight at 4°C in GDB buffer (0.2% BSA, 0.8 M NaCl, 0.5% Triton X-100, 30 mM phosphate buffer, pH 7.4). After 3 washes in PBS, secondary antibodies were incubated in the same buffer for 1hr at RT. Coverslips were mounted using Vectashield (Vectorlabs).

#### Uptake Experiment

Fixed neurons: Extracellular anti-Pan Neurofascin (1/200 in Neurobasal) was incubated with live neurons for 30 min at RT, the coverslips were then washed 2 times in warm Neurobasal, returned to original medium for 10min and fixed in warm paraformaldehyde (4%)-sucrose (4%) for 10min. Secondary anti-mouse Alexa-647 antibody diluted in 1/200 was incubated in PBS-NGS 5% for 30min at RT to stain the surface pool of NF186. Then cells were permeabilized in PBS-NGS 5%- Triton X-100 0.25% for 5min and blocked in PBS-NGS 10% for 30 min at 37degrees. Secondary anti-mouse Alexa 488 or −405 antibody was diluted 1/200 in PBS-NGS 5% and incubated for 30min at RT in order to stain the intracellular pool of NF186.

Live neurons: CF640R coupled (Mix-n-Stain, Biotium) extracellular anti-Pan Neurofascin antibody (1/200 in Neurobasal) was incubated with live neurons for 30 s at 37°C, the coverslips were then washed 1 time for 30 s in warm Neurobasal and original medium was added back before starting imaging.

#### Image Acquisition

Cells were imaged using a LSM700 confocal laser-scanning microscope (Zeiss) with a Plan-Apochromat 63x NA 1.40 oil DIC, EC Plan-Neofluar 40x NA1.30 Oil DIC and a Plan-Apochromat 20x NA 0.8 objective. Each confocal image was a z stack of 2–10 images, each averaged 4 times, covering the entire region of interested from top to bottom. Maximum projections were done from the resulting z stack. For fluorescence intensity comparison, settings were kept the same for all conditions.

*In vitro* assays were imaged on an iLas^2^ TIRF microscope setup as described (Sharma et al., 2016). In brief, ILas^2^ system (Roper Scientific, Evry, France) is a dual laser illuminator for azimuthal spinning TIRF illumination and with a custom modification for targeted photomanipulation. This system was installed on the Nikon Eclipse Ti-E inverted microscope with the perfect focus system, equipped with Nikon Apo TIRF 100x 1.49 N.A. oil objective (Nikon), EMCCD Evolve mono FW DELTA 512x512 camera (Roper Scientific) with the intermediate lens 2.5X (Nikon C mount adaptor 2.5X), CCD camera CoolSNAP MYO M- USB-14-AC (Roper Scientific), 150 mW 488 nm laser, 100 mW 561 nm laser and 49002 and 49008 Chroma filter sets and controlled with MetaMorph 7.8.8 software (Molecular Device). The final magnification using Evolve EMCCD camera was 0.064 μm/pixel and for CoolSNAP Myo CCD camera it was 0.045 μm/pixel. Temperature was maintained at 30°C to image the *in vitro* assays using a stage top incubator model INUBG2E-ZILCS (Tokai Hit). Time-lapse movies to estimate MT dynamics were acquired using a CoolSNAP Myo CCD camera (Roper Scientific), while movies for intensity analysis were acquired using a more sensitive Photometrics Evolve 512 EMCCD camera (Roper Scientific) at 2 s per frame with 100 ms exposure time for 10 minutes.

#### gSTED Microscopy

gated STED (gSTED) imaging was performed with a Leica TCS SP8 STED 3X microscope using a HC PL APO 100 × / 1.4 oil immersion STED WHITE objective. The 488, 594 and 647 nm wavelengths of pulsed white laser (80 MHz) were used to excite the Alexa488, Alexa594 and the Atto647N secondary antibodies. Both Alexa594 and Atto647N were depleted with the 775 nm pulsed depletion laser, Alexa488 was depleted with the 592 nm pulsed depletion laser (30%–40% of maximum power), and we used an internal Leica HyD hybrid detector (set at 100% gain) with a time gate of 0.3 ≤ t_g_ ≤ 6 ns.

#### Expansion Microscopy

Expansion microscopy was performed according to proExM protocol (Tillberg et al., 2016). Briefly, stained cells on an 18 mm coverslip were incubated overnight in 0.1 mg/mL Acryloyl-X (Thermo Fischer A20770) in PBS and 0.002% (solids) of 0.1 μm yellow-green FluoroSpheres (ThermoFisher, F8803). After washing three times in PBS, cells were transferred to gelation chamber (diameter 13 mm and 120 μL volume) made of silicone molds (Sigma Aldrich, GBL664107) on a parafilm covered glass slide. Chamber was pre-filled with monomer solution (2 M NaCl, 8.625% (w/w) sodium acrylate, 2.5% (w/w) acrylamide, 0.15% (w/w) N,N′-methylenebisacrylamide in PBS) with added 0.4% (w/w) tetramethylethylenediamine (TEMED) accelerator and 0.2% (w/w) ammonium persulfate (APS) initiator. The gelation proceeded for one 1 h at 37°C in a humidified incubator. Gels were further immersed into 2 mL of 8 units/mL proteinase-K in digestion buffer (50 mM Tris (pH 8), 1 mM EDTA, 0.5% Triton X-100, 0.08M guanidine HCl) solution for 4 h at 37°C for digestion. Gels were transferred to 50 mL deionized water for overnight expansion with water refreshed once to ensure the expansion reached plateau. Plasma-cleaned #1.5 coverslips were incubated in 0.1% (w/v) poly-L-lysine to reduce gel’s drift during acquisition. Gels on coated coverslips were mounted using custom printed imaging chambers [https://www.tinkercad.com/things/7qqYCygcbNU]. Expansion factor was calculated as a ratio of a gel’s diameter to the diameter of gelation chamber and was in the range of 4.2-4.4.

Confocal microscopy of expanded gels was performed with a Leica TCS SP8 STED 3X microscope using a HC PL APO 63x/1.20 W CORR CS2 water immersion objective. Images were acquired with lateral pixel size in the range of 70-80 nm and axial of 180 nm using internal HyD detector. If necessary, a drift correction of Z stack was performed in Huygens Professional version 17.04 (Scientific Volume Imaging, the Netherlands) using cross-correlation between adjacent slices. All images were deconvolved in the same program, using the CMLE algorithm, with SNR:7 and 20 iterations.

#### Single Molecule Localization Microscopy

Single Molecule Localization Microscopy was performed on a Nikon Eclipse Ti-E equipped with a 100x Apo TIRF oil immersion objective (NA 1.49) and Perfect Focus System 3. A Lighthub-6 (Omicron) with a 488nm laser (Luxx 200mW Omicron) and a 561nm laser (Coherent Obis) was used for excitation through a custom illumination pathway that allowed tuning of the incident angle. A quad-band polychroic mirror (ZT405/488/561/640rpc, Chroma), quad-band emission filter (ZET405/488/561/640 m, Chroma), and additional single-band emission filters were placed before the sCMOS camera (Hamamatsu flash 4.0v2) to separate the emission light from the excitation light. DIV4 neurons, nucleofected with mEos-tubulin and control-shRNA or AnkG-shRNA, were extracted with prewarmed 0.25% triton-X and 0.15% glutaraldehyde in MRB80 buffer for 1 minute. Subsequently, the samples were fixed with 4% paraformaldehyde in MRB80 buffer. After 3 PBS washing steps the samples were permeabilized with 0.25% triton-X in PBS for 10 minutes. After 3 more PBS washes, fiducial markers (FluoSpheres, Thermo Fisher) were added to enable drift correction, and the cells were mounted in a Ludin chamber in PBS. Upon identification of the axon initial segment, single-molecule localization microscopy of mEos-tubulin was performed with 100 ms exposure time, 561 nm laser excitation and low intensity 405 nm laser illumination to trigger photoconversion. Subsequently, IRIS (image reconstruction by integrating exchangeable single-molecule localization) was performed using LifeAct-mNeonGreen to image actin as described previously (Kiuchi et al., 2015, Schatzle et al., 2018, Tas et al., 2018). Briefly, LifeAct-mNeonGreen-6xHis in a pET28a vector was expressed in BL21DE3, purified using Complete His-tag purification resin (Sigma) and stored in PBS supplemented with 1mM DTT and 10% Glycerol. The purified protein was added to the fixed samples at such concentrations that single-molecule binding event could be detected at 100 ms exposure times. Reconstructions of the individual channels were performed using Detection of Molecules (DoM, https://github.com/ekatrukha/DoM_Utrecht).

#### Live Cell Imaging

Live-cell imaging experiments were performed in an inverted microscope Nikon Eclipse Ti-E (Nikon), equipped with a Plan Apo VC 100x NA 1.40 oil and a Plan Apo VC 60x NA 1.40 oil objective (Nikon), a Yokogawa CSU-X1-A1 spinning disk confocal unit (Roper Scientific), a Photometrics Evolve 512 EMCCD camera (Roper Scientific) and an incubation chamber (Tokai Hit) mounted on a motorized XYZ stage (Applied Scientific Instrumentation) which were all controlled using MetaMorph (Molecular Devices) software. Coverslips were mounted in metal rings and imaged using an incubation chamber that maintains temperature and CO_2_ optimal for the cells (37°C and 5% CO_2_). Neuron live imaging was performed in full conditioned medium and fresh medium was added to COS-7 before imaging.

Time-lapse live-cell imaging of EB3-RFP was performed with a time acquisition of 1 s. NF186-RFP alone was acquired at 2 frames per second. NPY-GFP, GFP-Rab6, Rab11-GFP or Rab5-GFP, alone or in combination with NF186-RFP were acquired at 10 frames per second.

For simultaneous imaging of green and red fluorescence, we used ET-mCherry/GFP filter set (59022; Chroma) together with the DualView (DV2; Roper) equipped with the dichroic filter 565dcxr (Chroma) and HQ530/30 m emission filter (Chroma).

#### MT Severing - Photoablation

Teem Photonics 355 nm Q-switched pulsed laser was used to perform laser-induced severing and study MT orientation in COS-7 cells and neurons as describe previously (Yau et al., 2014). No signs of toxicity to cells was observed during laser-induced severing.

#### Image Processing and Data Analysis

Movies and images were processed using Fiji (https://imagej.net/Fiji). Kymographs were generated using the ImageJ plugin KymoResliceWide v.0.4 https://github.com/ekatrukha/KymoResliceWide. Internalized NFasc vesicles were detected using the plugin ComDet (https://github.com/ekatrukha/ComDet). Fluorescence intensity of AIS proteins and AIS position were measured using the plugin Pro_Feat_Fit (https://github.com/cleterrier/Measure_ROIs). For the *in vitro* reconstitution assays, MT dynamics parameters viz. MT plus-end growth rate, catastrophe frequency, and rescue frequency were determined from kymographs using an optimized version of the custom made JAVA plugin for ImageJ as described previously (Mohan et al., 2013, Montenegro Gouveia et al., 2010, Smal et al., 2009). For TRIM46-decorated MT bundles, MT dynamics were quantified from the lagging MT within bundle and only two-MT bundles were selected for analysis. The relative standard error for catastrophe frequency and the relative standard error of mean for rescue frequency was calculated as described in (Sharma et al., 2016). One-way ANOVA with Tukey’s multiple comparisons test was performed to test for significance. For the quantification of parallel and anti-parallel MT-bundles fractions, polarity was determined by the velocity of MT polymerization. For intensity analysis of 480AnkG-mCherry on single MTs versus two-MT bundles, kymographs were generated with maximum intensity projection, and mean intensities on single MTs and two-MT bundles from the same TRIM46-positive MT bundles were estimated from multiple ROIs 1 μm^2^ in size. For each movie, individual mean intensities for single MTs or two-MT bundles after background subtraction were normalized to average mean intensity on single MTs quantified from the same movie and fold change in 480AnkG mean intensity were plotted for single MTs and dynamic and stable lattice in TRIM46-decorated two-MT bundle. Similarly, average mean intensity of GFP-TRIM46 on single MTs or two-MT bundles with or without 480AnkG was quantified from multiple ROIs 1 μm^2^ in size from assays done in two separate chambers under the same coverslip with identical acquisition settings. Pairwise mean comparisons between single MTs with and without 480AnkG and between two-MT bundles with and without 480AnkG were carried out using parametric two-tailed unpaired t test. Data are presented as mean ± SEM unless stated differently.

To measure microtubule-cell cortex distances, 0.1 μm large lines were traced perpendicular to the axons every 2 μm. Distances corresponding to a fixed intensity of 0.2 were extracted from the intensity profiles of each channel.

#### Electron Microscopy

To correlatively image COS-7 cells with fluorescence and electron microscopy we used a recently developed approach (Harterink et al., 2019). COS-7 cells were grown on needle engraved Aclar pieces (Electron Microscopy Sciences; 50426–10 (Jiménez et al., 2010))

glued in a 12-wells plate with Matrigel (Biosciences). Cells were transfected with NF186-RFP together with 480AnkG-GFP or 480AnkG-NN-GFP and extracellularly labeled with anti-NF186 (1:200; NeuroMab, A12/18). Cells were washed in culture medium and fixed with 2% paraformaldehyde + 0.2% glutaraldehyde in 0,1M PHEM buffer (pH 6.9) for 30 minutes. Free aldehyde groups were quenched with NH4Cl, washed with PBS, incubated with a bridging secondary antibody (1:300; Dakocytomation, Z0412) in PBS, 1% BSA, washed with PBS, 0.1% BSA and incubated with both protein-A gold 15 nm (1:60; CMC Utrecht) in PBS, 1% BSA and washed in PBS. Transfected cells were fluorescently imaged and the relative position to the engravings was documented. Cells were further fixed with 3.5% glutaraldehyde and 1% paraformaldehyde in 0.1 M cacodylate buffer (pH 7.4), post-fixed with a 1% Osmium and 1.5% KFeCN solution in 0,1 M cacodylate buffer (pH 7.4), washed with water, dehydrated with ethanol and infiltrated with increasing amounts of Epon resin. After polymerization, Aclars were peeled off leaving the target cells in the Epon. Landmarks were placed around the target cell using a microdissection setup (Zeiss, PALM microbeam) (Kolotuev et al., 2009) and a 3nm gold layer was applied using a sputter coater. Excess gold was wiped off using a cotton-stab and a drop of Epon was added on top of the cells and polymerized. The Epon samples were trimmed toward the target cell using the landmarks and sectioned with a Leica Utracut E microtome (60 nm). Sections were placed on grids (Cu 50M-H coated with Formvar film and carbon), stained with uranyl acetate and lead citrate and examined in a Tecnai10 or Tecnai12 electron microscope (FEI Company) operating at 100 kV and equipped with a SIS CCD Megaview II camera (Tecnai10) or a Tietz TVIPS TemCam F-214 (Tecnai12). Landmarks were used to identify the target cell.

#### EB1/2/3 KO Cell Line Generation

The CRISPR/Cas9 mediated EB1, EB2 and EB3 knockout was performed as previously described (Ran et al., 2013). In brief, U2OS cells were transfected with the vectors bearing the appropriate targeting sequences. One day after transfection, U2OS cells were subjected to selection with 2 μg/ml puromycin for 2 days. After selection, cells were allowed to recover in normal medium for ∼2 days, and knockout efficiency was checked by immunofluorescence staining. Depending on the efficiency, cells were isolated and characterized by western blotting and immunostaining. U2OS EB1/2/3 knockout cells were generated by targeting EB1, EB2 and EB3-encoding genes simultaneously. The pSpCas9-2A-Puro (PX459) vector that was used for the CRISPR/Cas9 knockout was purchased from Addgene. Guide RNAs for human EB1, EB2 and EB3 (also known as MAPRE1, MAPRE2, and MAPRE3) were designed using the CRISPR design webpage tool (http://zlab.bio/guide-design-resources). The targeting sequences for gRNAs were as follows (coding strand sequence indicated): EB1, 5′- TGGAAAAGACTATGACCCTG-3′; EB2, 5′- CCGGAAGCACACAGTGCGCG-3′ and EB3, 5′- TGCACCTCAACTATACCAAG-3′.

#### Protein Purification for *In Vitro* Reconstitution Assays

GFP-TRIM46, 480AnkG-GFP, 480AnkG-NN-GFP, 480AnkG-mCherry 480AnkG-NN-mCherry and GFP-PRC1proteins used in the *in vitro* reconstitution assays were purified using Strep(II)-Strep-Tactin affinity purification as described previously (Sharma et al., 2016). HEK293T cells were transfected with the constructs using polyethylenimine (PEI) and harvested 2 days post transfection. Culture medium was refreshed next day following transfection. Cells were lysed in cell lysis buffer (50 mM HEPES, 300 mM NaCl and 0.5% Triton X-100, pH 7.4) supplemented with protease inhibitor cocktail (Roche). Cell lysate was subjected to centrifugation at 14,800 rpm for 20 minutes at 4°C. The supernatant obtained from the previous step was incubated with Strep-Tactin Sepharose beads (GE) for 45 minutes. Following incubation, beads were washed with the lysis buffer without protease inhibitors 2 times and protein was eluted in 80 μl of elution buffer (50 mM HEPES, 150 mM NaCl, 1 mM MgCl2, 1 mM EGTA, 1 mM dithiothreitol (DTT), 2.5 mM d-Desthiobiotin and 0.05% Triton X-100, pH 7.4). Purified proteins were snap-frozen and stored at −80°C. Bacterially expressed mCherry-EB3 and dark EB3 were a gift of Dr. M.O. Steinmetz (Paul Scherrer Institut, Switzerland); they were produced as described previously (Montenegro Gouveia et al., 2010). Purity of the samples was analyzed via SDS-PAGE and Coomassie staining.

#### *In Vitro* Reconstitution Assays

*In vitro* reconstitution of MT dynamics was performed as described previously (Mohan et al., 2013). Guanylyl-(α,β)-methylene-diphosphonate (GMPCPP) stabilized MT seeds composed of 70% porcine tubulin, 18% biotin tubulin and 12% rhodamine-tubulin were prepared. Flow chambers were assembled using plasma-cleaned glass coverslips and microscopic slides. These chambers were then functionalized by incubation with 0.2 mg/ml PLL-PEG-biotin (Susos AG, Switzerland) followed by 1 mg/ml NeutrAvidin (Invitrogen) in MRB80 buffer (80 mM piperazine-N,N[prime]-bis(2-ethanesulfonic acid), 4 mM MgCl2, and 1 mM EGTA, pH 6.8). GMPCPP stabilized biotin-labeled MT seeds were attached to the coverslips through biotin-NeutrAvidin links. Flow chambers were further incubated with 0.8 mg/ml κ-casein to prevent non-specific protein binding. The reaction mix with or without proteins [MRB80 buffer supplemented with 15 μM porcine brain tubulin, 50 mM KCl, 1 mM guanosine triphosphate (GTP), 0.5 mg/ml κ-casein, 0.1% methylcellulose, and oxygen scavenger mix (50 mM glucose, 400 μg/ml glucose-oxidase, 200 μg/ml catalase, and 4 mM DTT)] were added to the flow chambers after centrifugation in an ultracentrifuge (Beckman Airfuge) at 119,000 × g for 5 minutes. All the experiments were done in the presence 20 nM of either mCherry-EB3 or dark EB3 when indicated. When added to the reaction mix, GFP-TRIM46 (20 nM), 480AnkG-GFP (12 nM), 480AnkG-NN-GFP (12nM) and 480AnkG-mCherry (10 nM) were used in the concentrations indicated in brackets. In the *in vitro* assays used for the intensity analysis of GFP-TRIM46 on the MT bundles, 0.5 μM rhodamine-tubulin was added to the reaction mix, while 14.5 μM porcine tubulin was used to make the final concentration of tubulin 15 μM. The flow chambers were then sealed with high-vacuum silicone grease (Dow Corning), and movies of these reconstitutions were acquired immediately at 30°C using a TIRF microscope. All tubulin products were from Cytoskeleton.

#### Mass Spectrometry

After streptavidin purification, beads were resuspended in 20 μL of Laemmli Sample buffer (Biorad) and supernatants were loaded on a 4%–12% gradient Criterion XT Bis-Tris precast gel (Biorad). The gel was fixed with 40% methanol/10% acetic acid and then stained for 1 h using colloidal Coomassie dye G-250 (Gel Code Blue Stain Reagent, Thermo Scientific). After in-gel digestion, samples were resuspended in 10% formic acid (FA)/5% DMSO and analyzed with an Agilent 1290 Infinity (Agilent Technologies, CA) LC, operating in reverse-phase (C18) mode, coupled to an Orbitrap Q-Exactive mass spectrometer (Thermo Fisher Scientific, Bremen, Germany). Peptides were loaded onto a trap column (Reprosil C18, 3 μm, 2 cm × 100 μm; Dr. Maisch) with solvent A (0.1% formic acid in water) at a maximum pressure of 800 bar and chromatographically separated over the analytical column (Zorbax SB-C18, 1.8 μm, 40 cm × 50 μm; Agilent) using 90 min linear gradient from 7%–30% solvent B (0.1% formic acid in acetonitrile) at a flow rate of 150 nL/min. The mass spectrometer was used in a data-dependent mode, which automatically switched between MS and MS/MS. After a survey scan from 350-1500 m/z the 10 most abundant peptides were subjected to HCD fragmentation. MS spectra were acquired in high-resolution mode (R > 30,000), whereas MS2 was in high-sensitivity mode (R > 15,000). Raw files were processed using Proteome Discoverer 1.4 (version 1.4.0.288, Thermo Scientific, Bremen, Germany). The database search was performed using Mascot (version 2.4.1, Matrix Science, UK) against a Swiss-Prot database (taxonomy human). Carbamidomethylation of cysteines was set as a fixed modification and oxidation of methionine was set as a variable modification. Trypsin was specified as enzyme and up to two miss cleavages were allowed. Data filtering was performed using percolator, resulting in 1% false discovery rate (FDR). Additional filters were search engine rank 1 and mascot ion score >20.

### Quatification and Statistical Analysis

All statistical details of experiments, including the definitions and exact values of n, and statistical tests performed, are shown in Figures and Figure Legends. n represent the number of analyzed cells, and N the number of independent experiments. Data processing and statistical analysis were done in Excel and GraphPad Prism (GraphPad Software). Significance was defined as: ns-not significant, ^∗^p < 0.05, ^∗∗^p < 0.01, and ^∗∗∗^p < 0.001. Normality of the data was determined by a D’Agostino and Pearson’s test and parametric two-tailed unpaired t test or non-parametric Mann Whitney test was applied. For more than one group, one or two-ways ANOVA were used followed by a Tukey’s multiple comparison test.

### Data and Code Availability

The mass spectrometry proteomics data have been deposited to the ProteomeXchange Consortium via the PRIDE partner repository with the dataset identifier PXD013685.

## Supplemental Information

Document S1. Figures S1–S7

Table S1. Mass Spectrometry Analysis of Purified GFP-TRIM46, 480AnkG-GFP, and 480AnkG-NN-GFP with a 10% Cutoff, Related to Figure 5

Document S2. Article plus Supplemental Information
