# Supplementary material for: Feedback-Driven Assembly of the Axon Initial Segment
Source: Neuron. 2019 Oct 23;104(2):305–321.e8. doi: 10.1016/j.neuron.2019.07.029 (PMC6839619; doi:10.1016/j.neuron.2019.07.029)
Supplement: Document S1. Figures S1–S7 [file mmc1.pdf]

Neuron, Volume *104*

Supplemental Information

Feedback-Driven Assembly

# of the Axon Initial Segment

Amélie Fréal, Dipti Rai, Roderick P. Tas, Xingxiu Pan, Eugene A. Katrukha, Dieudonnée van de Willige, Riccardo Stucchi, Amol Aher, Chao Yang, A.F. Maarten Altelaar, Karin Vocking, Jan Andries Post, Martin Harterink, Lukas C. Kapitein, Anna Akhmanova, and Casper C. Hoogenraad

Figure S1: 480AnkG recruits MTs to the plasma membrane

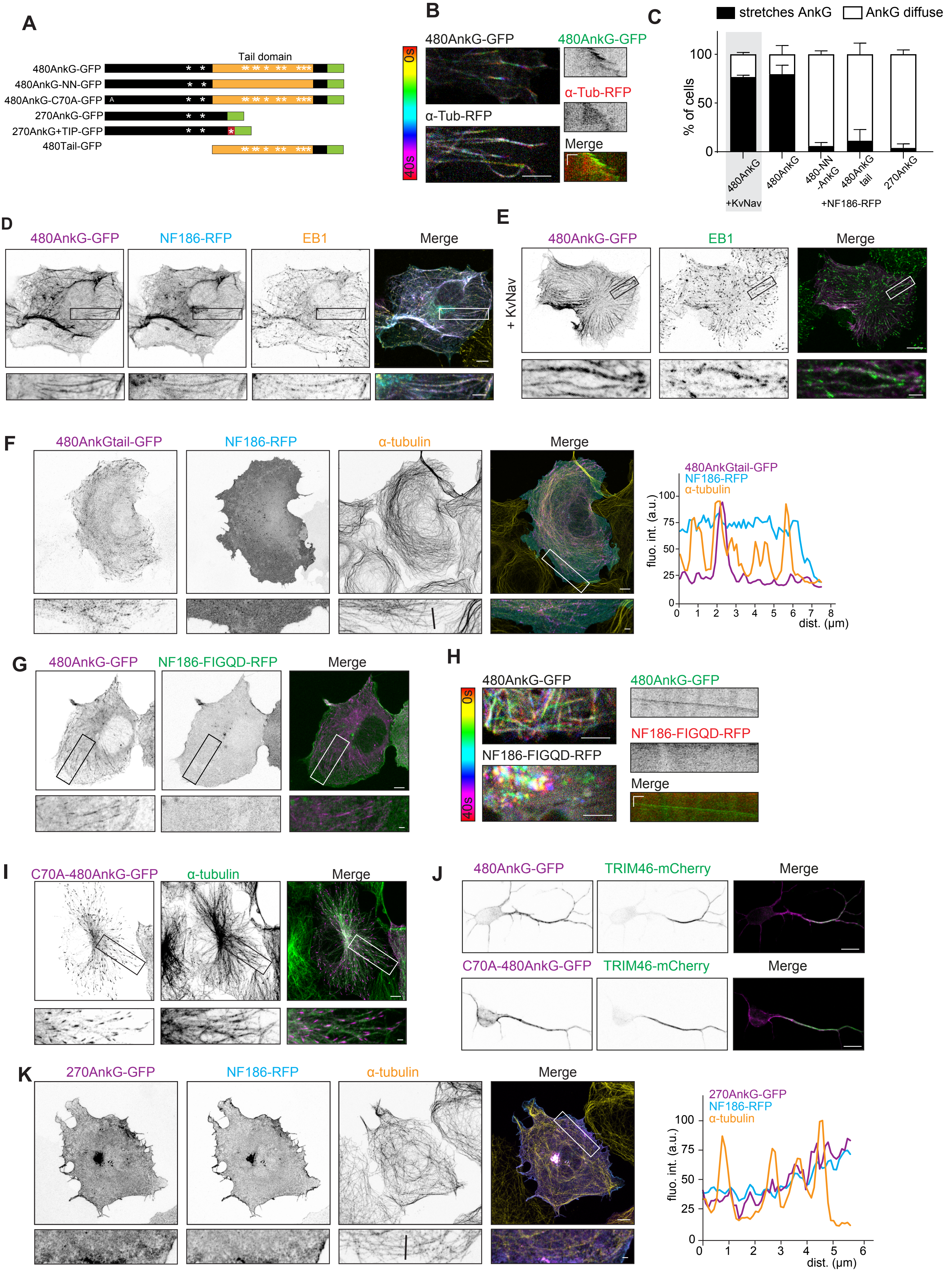

Figure S1. Related to Figure 1: 480AnkG recruits MTs to the plasma membrane

A. Scheme of AnkG constructs used in this study. The specific Tail of 480AnkG is shown in orange and GFP in green and asterisks show SxIP motifs.

B. Temporal-coded maximum projections from time lapse-imaging of a COS-7 cell expressing 480AnkG-GFP with α-Tub-RFP. Representative kymographs from this cell is shown on the right. Color-coded time scale is shown on the left.

C. Percentage of COS-7 cells transfected with indicated constructs showing AnkG as stretches (black) or diffuse (white). At least 150 cells were counted from 2 experiments for each condition.

D-E. COS-7 cells co-transfected with 480AnkG-GFP and either NF186-RFP (D) or Kv-Nav (E) and stained for GFP and EB1. Lower panels show zooms of the boxed areas.

F. COS-7 cell co-expressing NF186-RFP together with 480AnkGtail-GFP and stained for α-tubulin. Lower panel shows a zoom of the boxed area. Fluorescence intensity profiles along the black bar in the zoom are shown on the right.

G-H. COS-7 cells transfected with 480AnkG-GFP and NF186-FIGQD-RFP and stained for GFP (G). Lower panel shows zooms of the boxed areas. (H) shows the temporal-coded maximum projections from time lapse-imaging of a COS-7 cells transfected with 480AnkG-GFP and NF186-FIGQD-RFP. Representative kymographs from this cell is shown on the right.

I-J. COS-7 cell (I) transfected with C70A-480AnkG-GFP and stained for GFP and α-tubulin. (J) shows DIV3 hippocampal neurons transfected at DIV0 with TRIM46-mCherry and 480AnkG-GFP (upper panel) or C70A-480AnkG-GFP (lower panel) and stained for GFP.

K. COS-7 cell co-expressing NF186-RFP together with 270AnkG-GFP and stained for α-tubulin. Lower panel shows a zoom of the boxed area. Fluorescence intensity profiles along the black bar in the zoom are shown on the right.

Scale bars represent 10 μm and 2 μm in the zooms and in the kymographs: 1 μm (horizontal) and 15 s (vertical).

Figure S2: Membrane recruitment of MTs by 480AnkG is EB-dependent

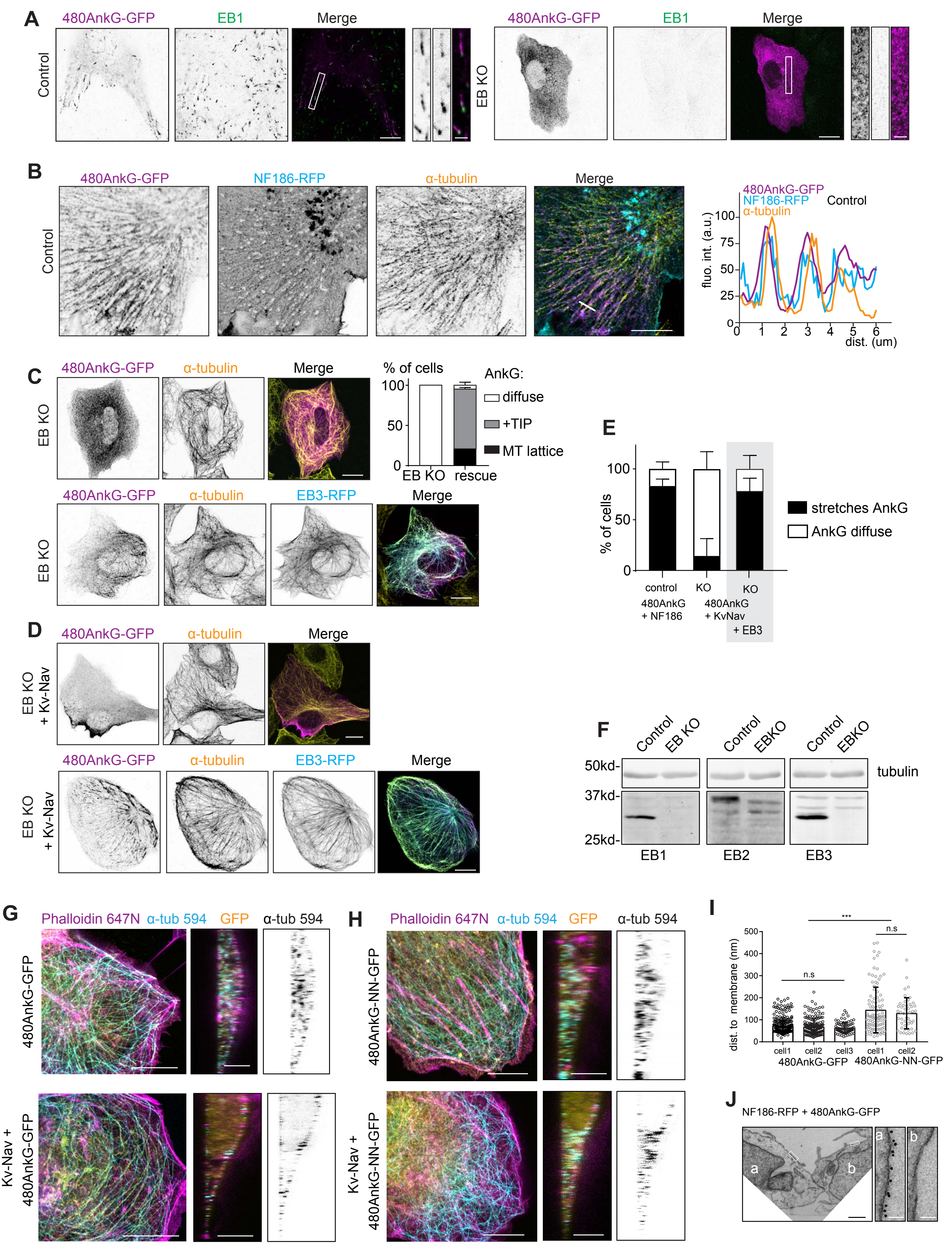

Figure S2. Related to Figure 3: Membrane recruitment of MTs by 480AnkG is EB-dependent

A. U2OS control (left panel) and EB1/2/3 KO (right panel) cells transfected with 480AnkG-GFP and stained for GFP and EB1. Zooms of the boxed areas are shown on the right of each panel.
B. U2OS control cell co-expressing 480AnkG-GFP and NF186-RFP, stained for α-tubulin. Fluorescence intensity along the white bar is shown on the right.
C-E. EB KO U2OS cells transfected with 480AnkG-GFP, with or without EB3-RFP (C) or co-expressing 480AnkG-GFP and KvNav with or without EB3-RFP (D) and stained for α-tubulin. Percentage of cells showing 480AnkG as stretches or as diffuse in indicated transfection conditions is shown in E.
F. Western-blot of U2OS EB KO and control cells lysates probed for EB1, EB2 and EB3. Tubulin was used as a loading control.
G-H. STED imaging of COS-7 co-expressing KvNav (lower panels) or not (upper panels) with 480AnkG-GFP (G) or 480AnkG-NN-GFP (H) stained for GFP, α-tubulin (Alexa595) and Phalloidin (Atto 647N). Z-sections are shown on the right.
I. Distance between MTs and membrane in COS-7 cells co-expressing NF186-RFP together with 480AnkG-GFP (3 cells) or 480AnkG-NN-GFP (2 cells). One-way ANOVA, n.s, $p>0.4$.
J. EM picture of COS-7 cells either co-expressing NF186-RFP and 480AnkG-GFP (left cell, a) or not transfected (right cell, b), which were immunogold labeled using an extracellular anti-NF186 antibody. No labeling could be observed on the non-transfected cell. Zooms of the indicated boxed areas are shown on the right.
In A-D, scale bars represent 10 μm, 2 μm in the zooms, in G-H, they represent 10 μm and 5 μm in the Z-sections. In J, scale bars represent 1 μm and 100 nm in the zooms.

Figure S3: TRIM46 localizes 480AnkG along the MT lattice

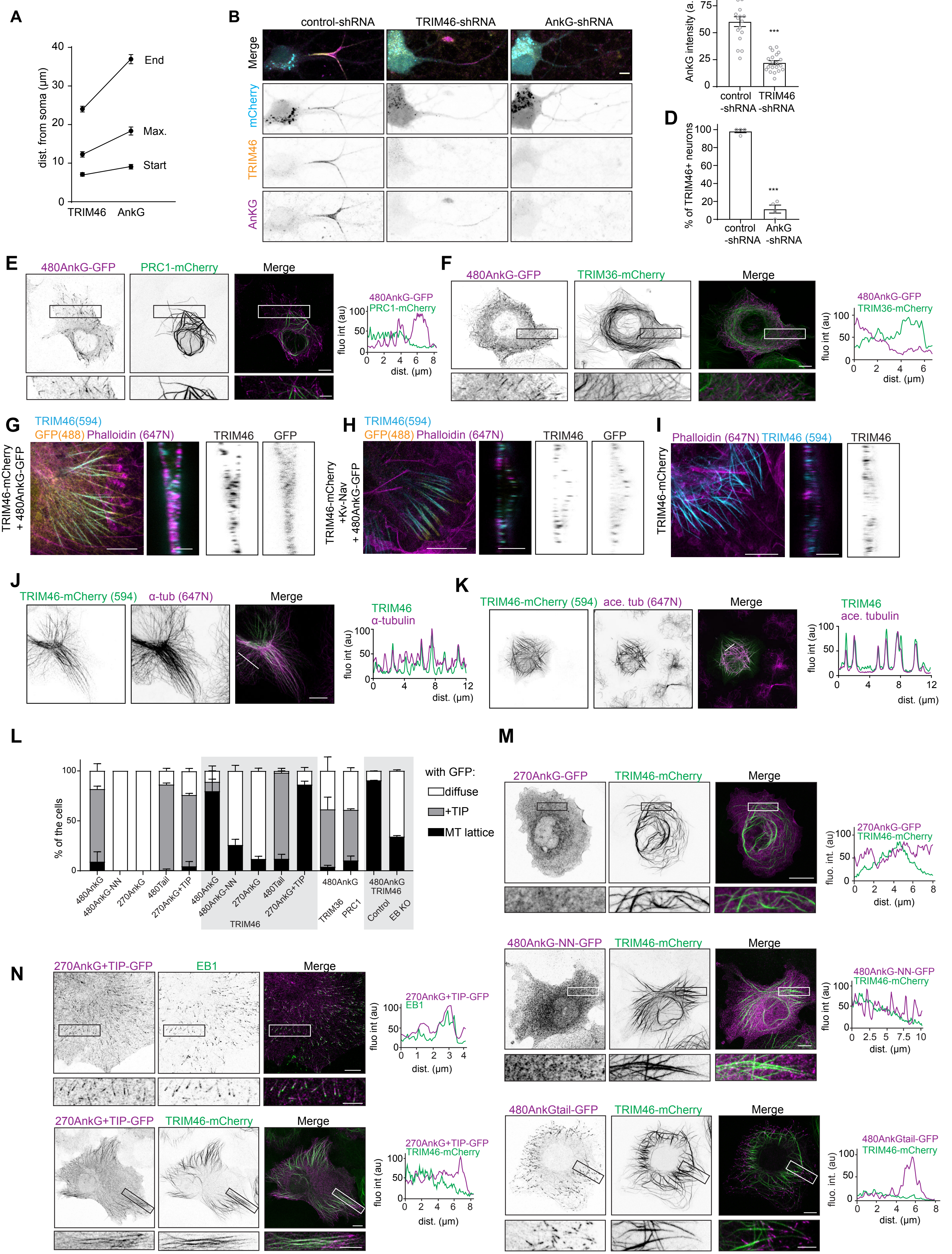

Figure S3. Related to Figure 3: TRIM46 localizes 480AnkG along the MT lattice

A-D. Mean distances from the soma of AnkG and TRIM46 starts, maxima and ends positions in DIV14 hippocampal neurons are shown in (A). n=32 neurons. (B) DIV14 hippocampal neurons cotransfected at DIV10 with a BFP-fill and control- (left), TRIM46- (middle) or AnkG-shRNA (left) and stained for TRIM46 and AnkG. (C) AnkG intensity at the AIS was measured in control and TRIM46-depleted neurons (at least 17 neurons from 2 experiments were analysed, unpaired t-test, ***$p<0.0001$). (D) Percentage of neurons showing TRIM46 immunoreactivity in the proximal axon in control conditions and upon AnkG depletion. (4 coverslips were counted for each phenotype, from 4 different cultures, unpaired t-test, ***$p<0.0001$).

E-F. C. COS-7 cells transfected with 480AnkG-GFP either with PRC1-mCherry (E) or with TRIM36-mCherry (F). Lower panels are zooms of the boxed areas and corresponding fluorescence intensity profiles are shown on the right.

G-I. STED imaging of COS-7 cells transfected with 480AnkG-GFP, TRIM46-mCherry (G) and Kv-Nav (I) and stained for GFP (Alexa488), TRIM46 (Alexa595) and Phalloidin (Atto 647N) or transfected with TRIM46-mCherry alone and stained for TRIM46 (Alexa 594) and Phalloidin (Atto 647N). Corresponding z-sections are shown on the right.

J-K. STED imaging of COS-7 cells expressing TRIM46-mCherry and stained for TRIM46 (Alexa594) and α-tubulin (Alexa647, J) or acetylated tubulin (Alexa647, K).

L. Percentage of cells expressing indicated constructs, showing GFP as diffuse (white bars), +TIP (grey bars) or on the MT lattice (black bars). At least 150 cells per condition from at least 2 experiments were counted.

M. COS-7 cells co-expressing TRIM46-mCherry together with 270AnkG-GFP (first panel), 480AnkG-NN-GFP (second panel) or 480AnkGtail-GFP (third panel). Lower panels are zooms of the boxed areas and corresponding fluorescence intensity profiles are shown on the right.

N. COS-7 cells expressing 270AnkG+TIP-GFP alone and stained for endogenous EB1 (first panel) or together with TRIM46-mCherry. Lower panels are zooms of the boxed areas and corresponding fluorescence intensity profiles are shown on the right.

Scale bars are 5 μm in B, 10 μm in E, F, M and N, and 2 μm in the corresponding zooms. They represent 10 μm in G-I and 5 μm in the Z-sections, 10 μm in J and 30 μm in K.

Figure S4: TRIM46 stabilizes and protects microtubules from depolymerization

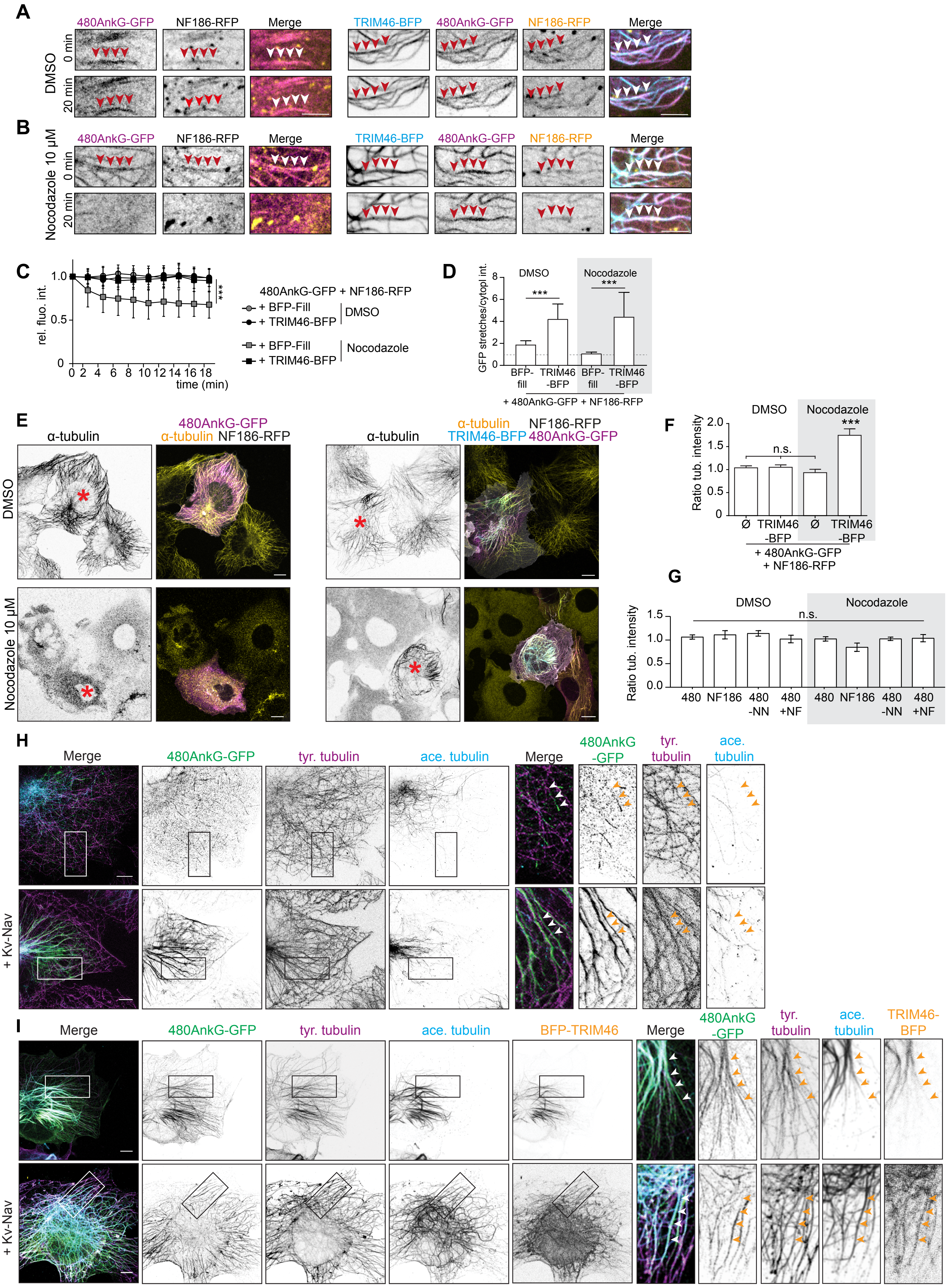

Figure S4. Related to Figure 4: TRIM46 stabilizes and protects MTs from depolymerization

A-D. Stills from time-lapse recordings of COS-7 cells expressing 480AnkG-GFP and NF186-RFP together with a BFP-fill (left) or with TRIM46-BFP (right), at 0 and 20 min after addition of DMSO (A) or Nocodazole (B, 10 μM). Arrowheads point at stable bundles.

Ratio of GFP fluorescence intensity along stretches over cytoplasm was measured over time after addition of DMSO or Nocodazole and normalized to the first frame (C) or measured after 20 min of the indicated treatment (D). Two-way ANOVA (C) and One-way ANOVA (D), ***$p<0.0001$, at least 19 ROIs were analyzed from at least 6 different cells.

E-G. COS-7 cells expressing NF186-RFP and 480AnkG-GFP (E, left panels) in combination with TRIM46-BFP (E, right panels) treated for 1 hr in DMSO 0.001% (upper panels) or in 10 μM Nocodazole (lower panels) and stained for α-tubulin. Asterisks indicate transfected cells. F and G show the α-tubulin fluorescence intensity ratio between transfected and non-transfected neighboring cells after indicated treatment. One-way ANOVA, 18-22 cells were analyzed per condition.

H-I gSTED imaging of COS-7 cells expressing 480AnkG-GFP (H, upper panel) in combination with KvNav (H, lower panel), or TRIM46-BFP (I, upper panel) or TRIM46-BFP and KvNav (I, lower panel) and stained for GFP (Alexa488), tyrosinated- (Alexa568) and acetylated tubulin (Alexa647) and TRIM46 (in I).

Scale bars represent 5 μm in A and B, 10 μm in E and 5 μm in H and J.

Figure S5: Specifity and EB-dependence of TRIM46-mediated enrichment of 480AnkG along MT bundles using *in vitro* reconstitution assays with purifed proteins

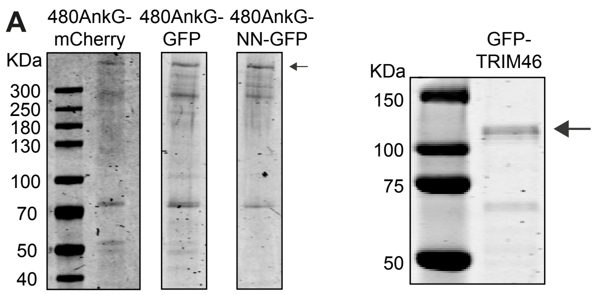

B

Identified proteins	GFP-TRIM46					480AnkG-GFP					480AnkG-NN-GFP				
	Score	PSMs	# Unique Peptides	# Peptides	Coverage	Score	PSMs	# Unique Peptides	# Peptides	Coverage	Score	PSMs	# Unique Peptides	# Peptides	Coverage
**TRIM46**	13713.88	418	56	56	62.19	441.79	15	9	9	16.21	190.49	5	5	5	10.67
**AnkG**						29955.88	958	141	157	32.65	27063.50	871	146	160	33.22
**EB1**	31.87	1	1	1	3.73	794.12	41	17	18	49.25	48.98	2	2	2	10.07
**EB2**	21.97	1	1	1	5.20										
**EB3**						183.41	13	10	11	34.16					
**Spectrin alpha chain**	221	9	9	9	4.65	4329.46	153	103	103	49.88	1894.52	81	70	70	35.23
**Spectrin beta chain**	150.59	5	5	5	2.88	2734.26	113	81	81	39.97	1421.36	63	50	50	23.98

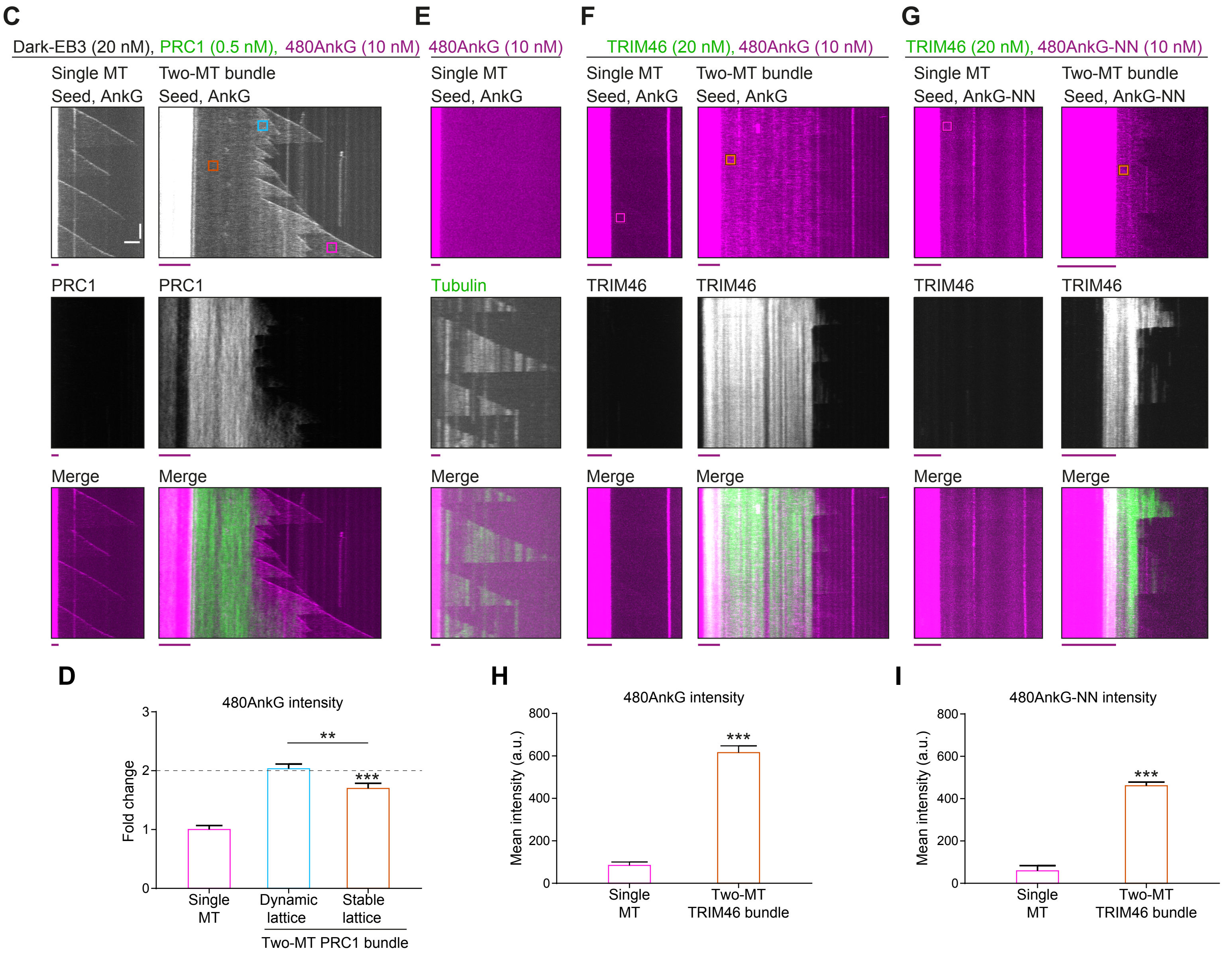

Figure S5. Related to Figure 5: Specificity of TRIM46-mediated enrichment of 480AnkG along MT bundles using in vitro reconstitution assays with purified proteins

A. Coomassie-blue stained gels with purified GFP-TRIM46, 480AnkG-GFP, 480AnkG-NN-GFP and 480AnkG-mCherry. Black arrows indicate isolated proteins.

B. Mass spectrometry analysis of purified GFP-TRIM46, 480AnkG-GFP and 480AnkG-NN-GFP. The table shows results for respective proteins and their major interacting partners, which were co-purified. Mass spectrometry results for other interactors and common contaminants have been included in the Table S1 with a top 10% cut-off.

C. Kymographs illustrating 480AnkG-mCherry intensity and dynamics of single MTs or PRC1 MT bundles grown *in vitro* in the presence of 20 nM dark-EB3, 0.5 nM GFP-PRC1 and 10 nM 480AnkG-mCherry. Colored boxes depict single MT (magenta), dynamic lattice (cyan) and stable lattice (orange) in two-MT bundle where ROIs were drawn to quantify 480AnkG-mCherry mean intensity.

D. 480AnkG-mCherry mean intensity on single MT or two-MT PRC1-positive bundles normalized to average mean intensity on single MTs. This data was obtained from 65-75 ROIs of 1 μm^2 in size from 15 PRC1-decorated two-MT bundles analyzed from 2 independent assays. Error bars represent ± SEM. One sample t-test was carried out to test if fold change in AnkG mean intensity on PRC1-positive MT bundles is more than 2, ***$p<0.001$. One-way ANOVA with Tukey's post-test was used to test if the change in 480AnkG intensity was different in dynamic compared to stable two-MT bundle, **$p=0.0032$.

E. Kymographs illustrating 480AnkG-mCherry fluorescence intensity on single MTs grown in vitro in the presence of 10 nM 480AnkG-mCherry, 14.5 μM unlabeled porcine tubulin and 0.5 μM HiLyte Fluor™ 488 labeled tubulin.

F-G. Kymographs illustrating 480AnkG-mCherry intensity and dynamics of single MTs or TRIM46 MT bundles grown in vitro in the presence of 20 nM GFP-TRIM46 and 10 nM 480AnkG-mCherry (F) or 10 nM 480AnkG-NN-mCherry (G) and in the absence of EB3. Colored boxes depict single MTs (magenta), and two-MT bundles (orange) where ROIs were drawn to quantify 480AnkG-mCherry or 480AnkG-NN-mCherry mean intensity. Since MTs were not labelled and the interaction of 480AnkG or 480AnkG-NN with MTs in the absence of EBs was weak, MT lattice regions were identified by proximity to MT seeds, where MTs outgrowth initiates.

H-I. 480AnkG-mCherry (H) and 480AnkG-NN-mCherry (I) mean intensity on single MT or two-MT TRIM46-positive bundles obtained from assays represented in (F) and (G) respectively. This data was obtained from 50 ROIs (H) and 15 ROIs (I) of 1 μm^2 in size from 5-10 single MTs or TRIM46-decorated two-MT bundles, analyzed from 2 independent assays. Error bars represent ± SEM. Pairwise mean comparisons between single MTs and two-MT bundles were carried out using two-tailed unpaired t-test, ***$p<0.001$.

Scale bars represent 2 µm (horizontal) and 60 s (vertical). The red and magenta lines below each kymograph represents rhodamine labeled GMPCPP-stabilized MT seeds.

Figure S6: NF186 travels via the endosomal pathway

Freal et al.

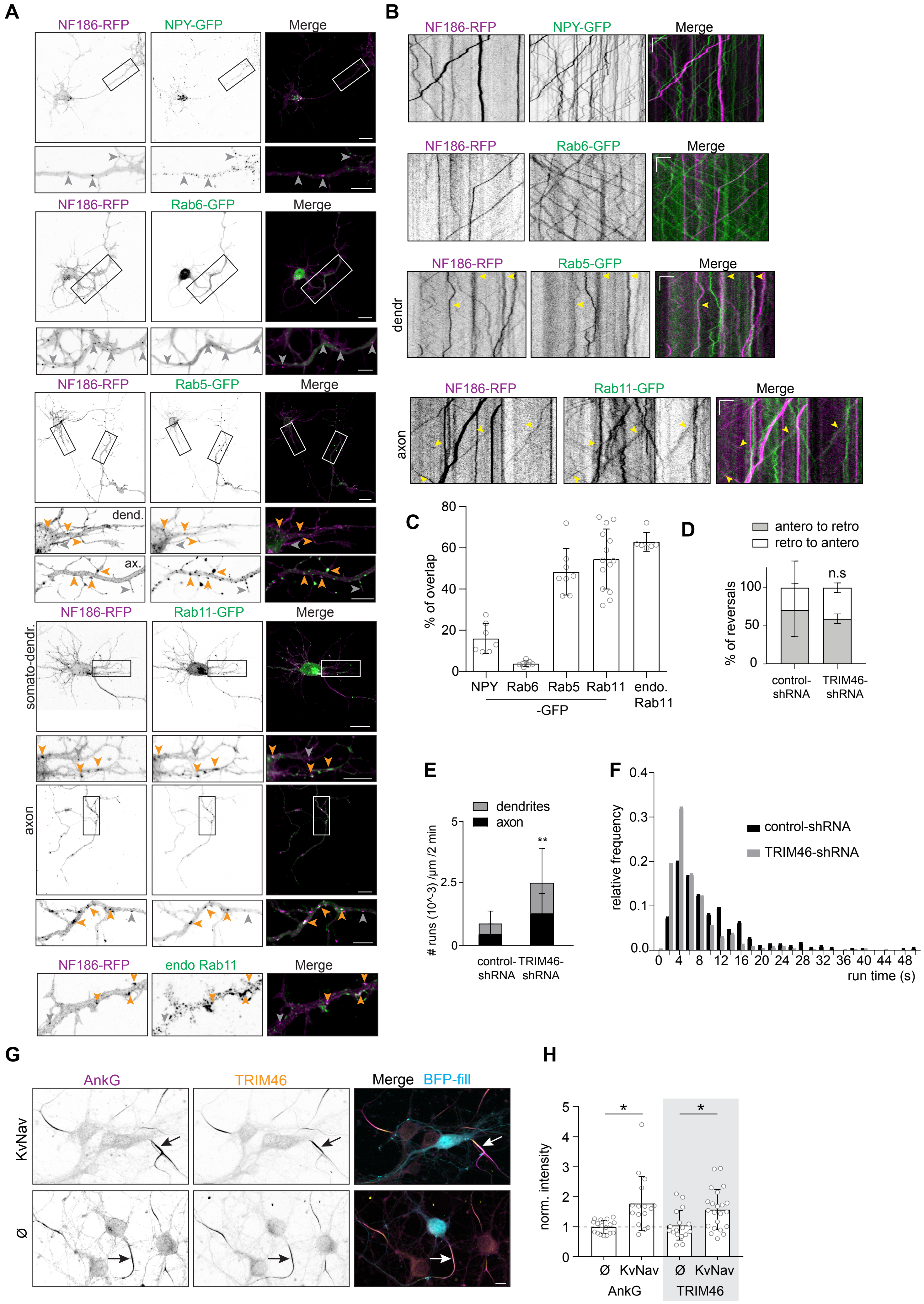

Figure S6. Related to Figure 6: NF186 travels via the endosomal pathway

A-C. Expression of NF186-RFP together with NPY-GFP, Rab6-GFP, Rab5-GFP or Rab11-GFP or stained for endogenous Rab11 in fixed (A) or live (B-C) DIV2 neurons. In A, orange arrowheads point to overlapping vesicles, whereas grey ones show vesicles only positive for NF186-RFP. Kymographs of NF186-RFP vesicles imaged together with NPY-GFP, Rab6-GFP, Rab5-GFP or Rab11-GFP are shown in B. The percentage of NF186-RFP vesicles colocalizing with the indicated markers in fixed neurons is shown in C. At least 440 NF186-RFP vesicles from at least 7 neurons from 2 experiments were counted.

D-E. Percentage of direction reversals from anterograde to retrograde (D, grey) or retrograde to anterograde (D, white) and number of runs (E) of NF186-RFP vesicles in neurons transfected with control- or TRIM46-shRNA. In D; n.s, p=0.43, two-way ANOVA, in E; p=0.013 in the axons, p=0.016 in the dendrites, two-way ANOVA.

F. Plot of the run times of NF186-RFP vesicles in neurons transfected with control- (black) or TRIM46-shRNA (grey).

G-H. DIV5 hippocampal neurons co-transfected at DIV1 with a BFP-fill and Kv-Nav (upper panel) or an empty vector (lower panel) and stained for AnkG and TRIM46. The intensity of AnkG and TRIM46 staining at the AIS normalized to neighbouring non-transfected neurons is shown in H. One-way ANOVA with Holm-Sidak's multpliple comparison test, p=0.011 for AnkG, p=0.037. At least 17 transfected neurons analyzed per condition, from 2 independent experiments.

In A, scale bars represent 20 µm and 10 µm in the zooms and 10 µm in G. In the kymographs, horizontal bar is 4.44 µm and vertical bar is 4 s.

Figure S7: AnkG allows for stable accumulation of NF186 at the AIS

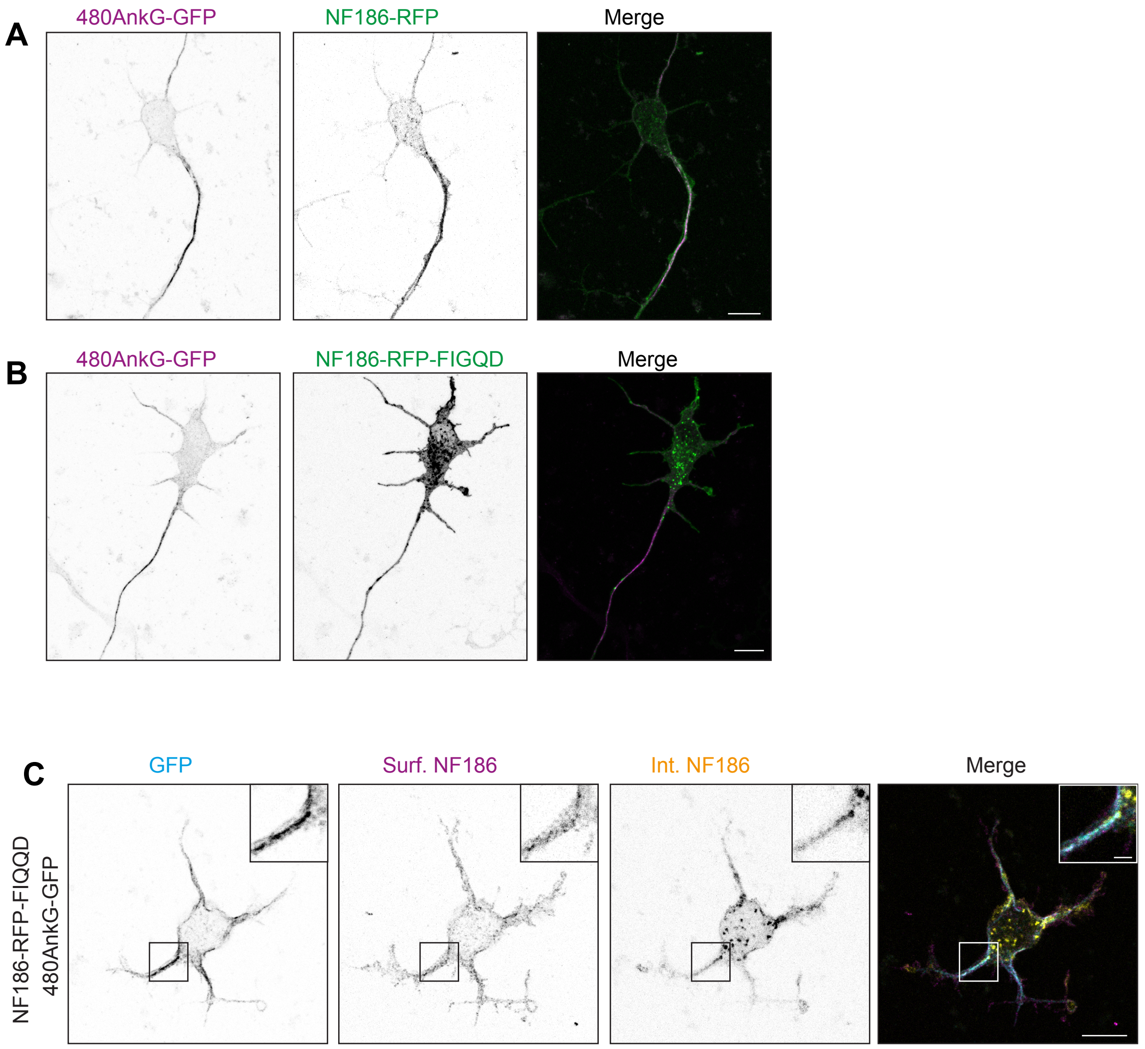

Figure S7. Related to Figure 7: AnkG allows for stable accumulation of NF186 at the AIS

A-B. DIV3 neurons transfected at DIV0 with 480AnkG-GFP and NF186-RFP (A) or NF186-RFP-FIGQD (B).
C. DIV1 neuron transfected at DIV0 with 480AnkG-GFP and NF186-RFP-FIGQD. Surface NF186 pool is shown in grey, and internalized pool is shown in red.
in A and B scale bars are 10 µm and in C they represent 10 µm and 2 µm in the zooms.
